# Uncoupled pyroptosis and IL-1β secretion downstream of inflammasome signaling

**DOI:** 10.3389/fimmu.2023.1128358

**Published:** 2023-04-06

**Authors:** Yang Li, Qianzhou Jiang

**Affiliations:** Department of Endodontics, Affiliated Stomatology Hospital of Guangzhou Medical University, Guangdong Engineering Research Center of Oral Restoration and Reconstruction, Guangzhou Key Laboratory of Basic and Applied Research of Oral Regenerative Medicine, Guangzhou, Guangdong, China

**Keywords:** ASC, caspase-1, death inflammasome, hyperactivation, inflammasome, inflammation, interleukin-1β, pyroptosis

## Abstract

Inflammasomes are supramolecular platforms that organize in response to various damage-associated molecular patterns and pathogen-associated molecular patterns. Upon activation, inflammasome sensors (with or without the help of ASC) activate caspase-1 and other inflammatory caspases that cleave gasdermin D and pro-IL-1β/pro-IL-18, leading to pyroptosis and mature cytokine secretion. Pyroptosis enables intracellular pathogen niche disruption and intracellular content release at the cost of cell death, inducing pro-inflammatory responses in the neighboring cells. IL-1β is a potent pro-inflammatory regulator for neutrophil recruitment, macrophage activation, and T-cell expansion. Thus, pyroptosis and cytokine secretion are the two main mechanisms that occur downstream of inflammasome signaling; they maintain homeostasis, drive the innate immune response, and shape adaptive immunity. This review aims to discuss the possible mechanisms, timing, consequences, and significance of the two uncoupling preferences downstream of inflammasome signaling. While pyroptosis and cytokine secretion may be usually coupled, pyroptosis-predominant and cytokine-predominant uncoupling are also observed in a stimulus-, cell type-, or context-dependent manner, contributing to the pathogenesis and development of numerous pathological conditions such as cryopyrin-associated periodic syndromes, LPS-induced sepsis, and *Salmonella enterica serovar Typhimurium* infection. Hyperactive cells consistently release IL-1β without LDH leakage and pyroptotic death, thereby leading to prolonged inflammation, expanding the lifespans of pyroptosis-resistant neutrophils, and hyperactivating stimuli-challenged macrophages, dendritic cells, monocytes, and specific nonimmune cells. Death inflammasome activation also induces GSDMD-mediated pyroptosis with no IL-1β secretion, which may increase lethality *in vivo*. The sublytic GSDMD pore formation associated with lower expressions of pyroptotic components, GSDMD-mediated extracellular vesicles, or other GSDMD-independent pathways that involve unconventional secretion could contribute to the cytokine-predominant uncoupling; the regulation of caspase-1 dynamics, which may generate various active species with different activities in terms of GSDMD or pro-IL-1β, could lead to pyroptosis-predominant uncoupling. These uncoupling preferences enable precise reactions to different stimuli of different intensities under specific conditions at the single-cell level, promoting cooperative cell and host fate decisions and participating in the pathogen “game”. Appropriate decisions in terms of coupling and uncoupling are required to heal tissues and eliminate threats, and further studies exploring the inflammasome tilt toward pyroptosis or cytokine secretion may be helpful.

## 1 Introduction

Inflammasomes are potent regulators of innate immunity that act as the first line of defense in response to numerous damage-associated molecular patterns (DAMPs) and pathogen-associated molecular patterns (PAMPs), thereby shaping adaptive immunity (1). Canonical inflammasomes are supramolecular organizing centers (SMOCs) that comprise a hierarchical architecture, with the sensor, the apoptosis-associated speck-like protein containing a caspase recruitment domain (ASC), and the executor (mainly caspase-1), leading to Gasdermin D (GSDMD)-mediated pyroptotic cell death and IL-1β and IL-18 secretion (2–4). The structures and functions of nucleotide-binding oligomerization domain (NOD)-like receptors (NLRs) such as NLRP3, NLRC4, NLRP1, and NLRP6, absent in melanoma 2 (AIM2)-like receptors (ALRs) and pyrin, have been reviewed elsewhere (5–10); they are common canonical platforms for the activation of caspase-1 and the maturation of its substrates. The non-canonical caspase-11/-4/-5 play pivotal roles in sensing intracellular LPS, inducing pyroptosis and non-canonical cytokine secretion in a caspase-1-dependent manner (11). Two downstream events, pyroptosis and cytokine secretion, serve as key weapons in maintaining homeostasis and protecting against infection, stress, and damage under various pathological conditions such as COVID-19, cancers, and Alzheimer’s disease (12–17). While pyroptosis and cytokine secretion may be usually observed as coupled events, evidence has shown that this scale can tip under the influence of inflammasome signaling with unclear mechanisms (18). This review aims to discuss recent advancements and provide insights into the pyroptosis-predominant and cytokine-predominant uncoupling propensities that lie downstream of inflammasome signaling. Overall, pyroptotic death and cytokine secretion are introduced, the association between ASC-mediated inflammasome assembly and caspase activation is traced, and the significance of these uncoupling preferences in physiological and pathological conditions is discussed. Our review will help future research in better understanding the inflammasome tilting toward pyroptosis or cytokine secretion.

## 2 Pyroptosis downstream of inflammasome signaling

Pyroptosis, a type of regulated cell death, is mainly mediated by gasdermin family members and is induced by transmembrane pore formation *via* the active N-terminal fragments, leading to cell swelling, plasma membrane rupture, and intracellular content release (19). Pyroptosis eliminates pathogen niches, exposes microbes, or maintains pathogens within pyroptotic corpses to propagate local inflammatory response (20–22). Pyroptotic pores and cell lysis also enable the release of certain intracellular cytokines and contents into the extracellular environment (23), facilitating the transferal of other immune sentinels to the point of injury or infection (24). Pyroptosis, therefore, plays a key role in homeostasis and host defense.

GSDMD, encoded by the gene *GSDMD* on chromosome 8q24.3, is the main executor of inflammasome-driven pyroptosis (25); it is widely expressed in various tissues (e.g., the colon, liver, and brain) and immune cells (26–28). GSDMD is exclusive to the mammalian genome (26). GSDMD-mediated pyroptosis has been observed in immune cells (29–31) such as macrophages and monocytes and in nonimmune cells (32–34) such as epithelial cells and osteoblasts. Upon inflammasome activation, canonical caspase-1 and noncanonical caspase-4/-5 (in humans)/-11 (in mice) are activated, cleaving GSDMD at D275 (FLTD|GVP in humans) or D276 (LLSD|GID in mice) and separating its active 31kDa N-terminal (GSDMD-NT) from its 22kDa autoinhibitory C-terminal fragments (35–37). GSDMD-NT interacts preferentially with negatively-charged membrane lipids, such as phosphatidylserine in the inner leaflet of the cell membrane and cardiolipin in the inner and outer leaflets of bacterial membranes; here, it oligomerizes into a ring-like structure that targets cell plasma, mitochondria, nucleus, and bacterial membranes, increasing the permeability and leading to intracellular architecture loss (38–40). The events between GSDMD cleavage and membrane pore formation could be controversial, because biophysical studies may have difficulties in determining whether GSDMD-NT monomers assemble into pore-forming oligomers before or after inserting into membranes. Recent evidence may suggest the latter, because membrane-associated GSDMD-NT monomers form oligomers when treated with ROS-inducing agents, while GTPases RagA and RagC are required in GSDMD-NT oligomerization but not in GSDMD-NT plasma membrane localization (41). The phospholipid phosphatase PtpB secreted from *Mycobacterium tuberculosis* (Mtb) can dephosphorylate cell membrane lipids to inhibit the membrane trafficking of GSDMD-NT (42). Therefore, membrane interaction and oligomerization are critical steps that are regulated by both the host and pathogens. GSDMD can also be cleaved by neutrophil elastase and cathepsin G to promote its pore-forming ability; it can be cleaved by caspase-3/-7 to generate an inactive p43 fragment and by caspase-8, which is driven by TAK1 inhibition in *Yersinia* infection at the same site as the inflammatory caspases in mouse macrophages (43–47). Other gasdermin family members such as GSDMA3, GSDMB, and GSDME can also form pores in cell membranes in an inflammasome-independent manner (48–50). Herein, we focus on the role that GSDMD plays in pyroptosis downstream of inflammasome signaling.

The exact structure and functionality of GSDMD pores remain elusive. GSDMA3, which is highly similar to GSDMD, organizes pore structures comprising 26–28 protomers that are 70 Å in height, with inner and outer diameters of 180 Å and 280 Å, respectively. These pores are likely to allow the cytokines IL-1β and IL-18, with diameters of around 4–8 nm (51, 52) to be released. GSDMA3 and GSDMD are thought to assemble prepores or prepore-like structures (arcs, slits, and rings) that form the functional larger pores (49, 53, 54). The 33 (varying between 31–34)-subunit GSDMD pore is characterized by a height of 80 Å and inner and outer diameters of 215 Å and 310 Å, respectively, and features a globular domain (more membrane-distal than GSDMA3 by approximately 16°) on the cytosolic side with a large transmembrane β-barrel (53). More precisely, four solvent-exposed acidic patches (AP1 and 4 in the globular domain, and AP2 and 3 in the β-barrel) are found near the conduit, contributing to negative potentials in the pore passage (53). GSDMD pore formation is regulated at different levels: autoinhibition, proteolytic cleavage, membrane lipid selectivity, and oligomerization (55–57). The presence of this conduit facilitates IL-1β and IL-18 secretion (discussed later) and ion passage, and the flux of K^+^ ions through these pores as a result of caspase-11 or AIM2 activation may act as a secondary signal for NLRP3 inflammasome activation (58–60). The flux of K^+^ ions through GSDMD pores in AIM2 inflammasome signaling may also restrict the response of the cGAS-dependent type I interferon (IFN) or promote the nonclassical release of IFN-β, indicating an additional role for GSDMD (61, 62). In contrast, the increased influx of Ca^+^ ions through the GSDMD pores may aid in membrane repair by recruiting endosomal sorting complexes required for transport (ESCRT) and removing damaged membranes that contain GSDMD pores from macrophages and HeLa cells (63). These results indicate a pro-inflammatory and pro-death role as well as the association of positive and negative feedback loops with GSDMD pores in inflammasome regulation.

**Figure 1:**
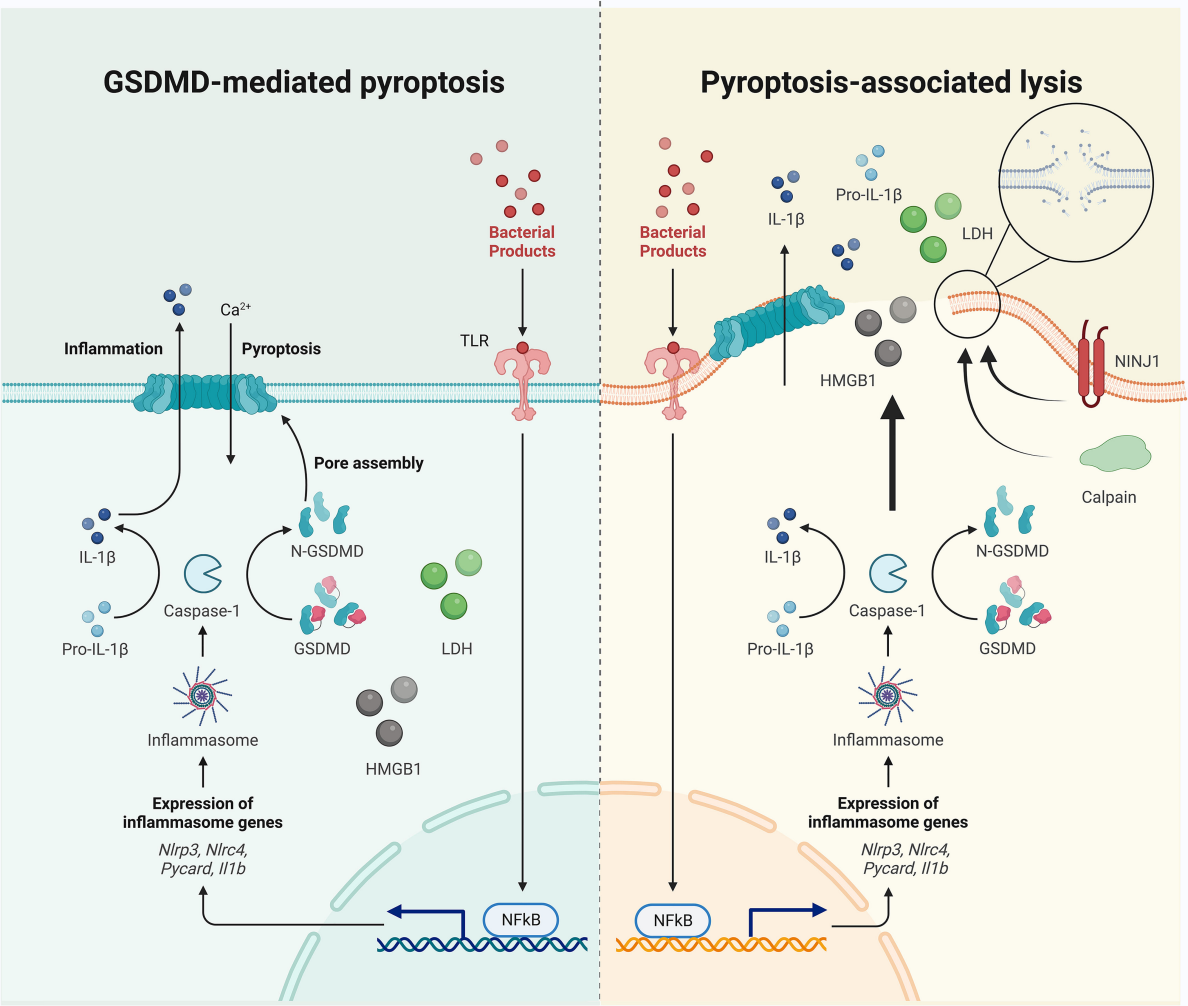
Pyroptotic cell death occurs before cell lysis. The separation of GSDMD pore formation and cell membrane rupture are evidenced by the differential release mechanisms of intracellular contents: IL-1β is secreted through GSDMD pores and ruptured cell membranes, and HMGB1 and LDH leak (if not always) *via* cell lysis. Cell swelling and plasma membrane rupture could be active events mediated by calpain and NINJ1, although unbalanced osmotically permeability induced by GSDMD pore formation may also contribute to passive lysis.

Under the increased intracellular pressure that follows ion and water flux, the pyroptotic cell may undergo swelling, rupture, and lysis, allowing for the extracellular release of relatively larger intracellular components that can act as DAMPs. Lactate dehydrogenase (LDH, diameter 8–10 nm) release is therefore regarded as a classic readout of lytic cell death (64). However, pyroptotic cell death can be separated from cell lysis (**Figure 1**). LDH release is inhibited by an anti-lytic agent glycine in bone marrow-derived macrophages (BMDMs), challenged by RodTox, an NLRC4 activator, and detected once the glycine is removed (65). In provoked BMDMs that express fluorophores, the loss of cytosolic tdTomato and GFP occurs 4–5 min after the first Sytox internalization, which can enter cells without intact plasma membranes and is interpreted as indicating the increased permeability that is initiated by GSDMD pore formation in this study. Glycine inhibits LDH leakage but cannot delay the loss of tdTomato or GFP, although their kinetics are slowed (65). More importantly, when Sytox enters the cell, cell movement stops, mitochondrial activity slows, and the cell starts to swell; these effects cannot be abolished by glycine (65). These results indicate that the GSDMD pore-mediated membrane permeability starts before cell lysis. Another independent study showed that mitochondrial membrane depolarization, Ca^2+^ internalization, phosphatidylserine exposure, cell swelling, and lysosome decay occur 18–21 min, 12–15 min, 9–12 min, 13 min, and 6–9 min, respectively, before PI or Sytox enter the cell (which is interpreted as cell membrane damage in this study) in NLRP1b signaling, and approximately 20 min, 6–9 min, 3 min, 13 min, and 6–9 min before PI or Sytox enter the cell in NLRC4 signaling (66). In contrast, nuclear rounding and condensation occur concurrently with the loss of cell membrane integrity (66). Despite the different interpretations, these results show that pyroptotic cell death closely coincides with GSDMD-mediated cell permeability, which occurs earlier than cell lysis, indicating that the inhibition of cell lysis may not block pyroptotic cell death. In line with these data, a recent study reported that deficiency of the cell-surface protein NINJ1 in BMDMs under LPS electroporation or nigericin treatment results in impaired pyroptotic cell membrane rupture and LDH release, while this deficiency does not significantly influence IL-1β secretion, suggesting that NINJ1 functions downstream of the GSDMD pores as LDH is only released during lysis (67). Ninj1^–/–^ macrophages die with ballooned morphology rather than a lytic appearance, indicating that pyroptotic cell death differs considerably from cell lysis (67). The separate events of GSDMD pore formation and cell membrane rupture are also evidenced by the differential release styles of intracellular contents; IL-1β, IL-18, and rhoGTPase Rac1 (5 nm) are secreted through GSDMD pores (often accompanied by detectable PI intake) and ruptured cell membranes, and HMGB1 (7.9 nm), caspase-1 heterotetramer (6.8 nm), and LDH leakage occur only as a result of cell lysis (52, 68). In contrast, one study found that glycine cannot inhibit the release of LDH or HMGB1 from pyroptotic THP-1 cells and that the swollen and unruptured cells release some pyroptotic contents such as IL-1β, LDH, and HMGB1, rendering the use of LDH and HMGB1 release as readouts for lysis suspicious (20). In addition, cell swelling could be an active process that is regulated by Ca^2+^ (from GSDMD pores and/or intracellular lysosomal leakage)-dependent calpain-driven vimentin cleavage and the consequent loss of intermediate filament (20). The cell rupture that occurs under the associated shear stress and compressive force enables the release of ASC specks and other macro-DAMPs (20). The authors further suggest that intracellular soluble microDAMPs (as large as 200 kDa) could escape through the GSDMD pores, while larger or nonsoluble macroDAMPs require cell rupture for release (20). Together, GSDMD pores could thus act as a filter that determines which “small” molecules can pass before cell lysis.

However, not all small molecules can be easily secreted through GSDMD pores. Pro-IL-1β, which is similar in size to IL-1β, is not secreted *via* GSDMD pores, indicating that GSDMD pores may act as filters that repel the precursor (53). This observation leads to the hypothesis that the negatively charged GSDMD pores preferentially allow the release of positively (e.g., IL-1β, which basifies during maturation) and neutral charged, as compared to negatively (e.g., pro-IL-1β, which has an acidic domain) charged, molecules. To support this assumption, mutations that diminish the negative potential of GSDMD pores markedly increase the release of negatively charged small dextrans (40 kDa) (53). Extrinsic factors (e.g., lipid and salt) may also affect the electrostatic environment of GSDMD pores and IL-1 transport (69). These data indicate that along with size, the charge of a cargo molecule is also important for passing through GSDMD pores. Notably, both anionic and cationic ultrasmall (<10 nm) nanoparticles could enter the pyroptotic macrophages through the GSDMD pores *via* passive diffusion and in a microtubule-independent way (70). GSDMD-dependent membrane perforation may also allow access for extracellular nanobodies targeting ASC to inhibit further inflammasome activity without affecting initial and pre-pyroptotic IL-1β secretion (71). These evidence may lead to the hypothesis that GSDMD pores may act as useful windows or passages for diagnostic and therapeutic strategies associated with extracellular drugs targeting pyroptotic cells. Collectively, these studies exhibit the discrepancies between active and passive swelling and rupture, and GSDMD pore-mediated and membrane rupture-mediated extracellular release of cell contents, exhibit the significance of different mechanisms of intracellular content release. Notably, the formation of GSDMD pores could occur on a sublytic level, avoiding cell rupture and LDH release while preserving IL-1β secretion (72).

Interesting questions, therefore, arise: what are the exact criteria by which “microDAMPs” and “macroDAMPs” are distinguished, and is the difference dependent on cell type (e.g., BMDMs versus THP-1 cells)? Is there a structural mode [e.g., the formation of large pores from GSDMD-NT prepores or prepore-like structures as mentioned previously, resembling the Bax pore formation on the mitochondrial outer membrane (73, 74)] resulting in early-stage ion-selectivity and late-stage non-selectivity in GSDMD pores, which may be independent of any subsequent cell membrane rupture? Would charge-based modification act as an effective strategy for developing new drugs? Further studies are required to explain the underlying mechanisms of GSDMD-mediated pyroptosis downstream of inflammasome signaling.

## 3 Cytokine maturation and secretion downstream of inflammasome signaling

IL-1β, a potent pro-inflammatory cytokine in the IL-1 family, is induced mainly in myeloid cells and some non-myeloid cells (e.g., epithelial cells), but it is not always constitutively expressed (12, 75). Its precursor is inactive and needs processing to generate a bioactive mature form (76). Hence, the production of IL-1β requires two steps in most cells: a priming signal (e.g., TLR ligation) for precursor expression, and a processing procedure for maturation. Caspase-1, the main effector caspase in canonical inflammasomes, cleaves pro-IL-1β, removing the pro-peptides and creating the mature form, while caspase-4/-11 are ineffective and caspase-5 has a weak ability in processing the precursor (12, 77, 78). IL-1β may also be processed by non-caspase-1 proteases such as caspase-8, proteinase-3, elastase, granzyme A, matrix metalloprotease 9, or chymase (79). However, IL-1β lacks a signal peptide for its release into extracellular space *via* the classical ER/Golgi pathway (80–82). Such a poorly understood release mechanism is denoted “unconventional protein secretion (83)”, for which several differential mechanisms and multiple models have been proposed. Upon inflammasome activation, mature IL-1β is released, both from GSDMD pores and passively *via* pyroptotic lysis, which are regarded as non-vesicular pathways. Intracellular mature IL-1β is detected in murine macrophages that have been primed with LPS (1 μg/ml, 2 h) and treated with ATP for 5 min, along with a detectable level of extracellular IL-1β in the supernatant (84). The amount of mature IL-1β within the cell lysates subsequently decreases from this point, correlating with an increase in the supernatant (84). LDH release is significantly enhanced after 10 min treatment with ATP (approximately 5 min after extracellular IL-1β detection), indicating that IL-1β release is not just a non-specific process in cell lysis, but also occurs in dying cells before rupture, most likely through GSDMD pores as mentioned previously (84). Mature IL-1β can accumulate within the cytosol of GSDMD^-/-^ macrophages, further indicating that the IL-1β maturation and GSDMD pore formation could act as parallel events during inflammasome activation (25). Besides the non-vesicular model, IL-1β may also be secreted in a vesicular manner that is dependent on GSDMD but not on the formation of pores, although other vesicular secretion models such as secretory autophagy, microvesicle shedding, multivesicular bodies and exosomes, and secretory lysosomes have also been proposed (52, 75, 85). The different unconventional protein secretion pathways of IL-1β *via* plasma membrane pores or vesicular carriers are closely dependent on cell types and the type and strength of stimulus (76, 86). Metabolic condition in host cells may also affect the secretion route of IL-1β, with mechanisms of pore-formation and cell lysis in nutrient repletion versus autophagic capture and vesicle intermediate translocation during starvation and ER stress (87–89). Notably, the polybasic motif may help direct mature IL-1β to the inner face of the plasma membrane for colocalization with PIP2 (perhaps indirectly), and this IL-1β enrichment may act as a prerequisite for its subsequent release *via* both GSDMD pore-dependent and independent mechanisms (90). Interestingly, the discrepancies between GSDMD-associated pyroptosis and unconventional IL-1β secretion may contribute to their uncoupling downstream of inflammasome signaling.

IL-1α, another form of IL-1, may also be released during inflammasome activation. Unlike IL-1β, active IL-1α precursor is constitutively expressed in numerous cells of different organs such as the kidney, liver, and lung, and is processed by elastase, calpain, granzyme B, and thrombin into a cleaved form with increased activity and receptor affinity (91–94). Human recombinant pro-IL-1α is cleaved by caspase-5, but not by caspase-1/4 (95), and murine IL‐1α is not processed by murine caspase-1 (95). The release of cleaved IL-1α from human and murine macrophages primed with LPS and transfected with intracellular LPS requires the presence of caspase-5 and caspase-11, respectively (95). Caspase-1 may indirectly promote IL-1α release by cleaving cytosolic IL-1R2, which binds to pro-IL-1α to prevent cleavage by calpain, thus dislocating pro-IL-1α for further processing in macrophages (96). IL-1α may shuttle to the nucleus to act as a transcription factor regulating gene expression (e.g., IL-8), or bind to the cell membrane receptor complexes IL-1R1/IL-1R3, sharing a proinflammatory function with IL-1β (12, 97). Compared to IL-1β, IL-1α is more likely secreted as a key alarm in cell lysis during pyroptosis, necrosis, and necroptosis (85). IL-1α has also been detected on cell membranes, indicating a possible association with the inadvertent permeabilization and leakage of IL-1α within the cell, and cell surface IL-1α could be further cleaved and released (98). IL-1β and IL-1α, together termed IL-1, could promote innate immunity by inducing CXC- and CCL- chemokines (e.g., IL-8) for neutrophil recruitment (99–101), directly [e.g., in myocardial infarction (102)] or indirectly [e.g., by enhancing TH17 cell differentiation (103–105)] upregulating granulopoiesis and mature neutrophil release from the bone marrow, increasing the formation of neutrophil extracellular traps (NETs) for the trapping and killing of bacteria (106–108), and driving M1/M2 polarization and macrophage activation (109–111). Some of these mechanisms [e.g., NET formation (112) and M1/Th1 activation (113)] could further amplify IL-1 production. IL-1 signaling also plays a vital role in shaping adaptive immunity by increasing dendritic cell (DC) maturation and chemokine secretion (114–116), directly or indirectly promoting T cell expansion, differentiation, and survival (117–119), and enhancing Tfh-mediated B cell proliferation and antibody production (120–122). In addition, IL-1 is critical in maintaining the epithelial barrier as epithelial cells encounter various PAMPs and DAMPs under both physical and pathological conditions (123–125). These results render IL-1 an important pro-inflammatory regulator in infectious, autoinflammatory, and autoimmune diseases such as HIV (126), COVID-19 (127–129), cancers (130–132), diabetes (133–135), and rheumatoid arthritis (136).

Another IL-1 family member, pro-IL-18, is processed by caspase-1/-4 into its mature form during inflammasome activation and released *via* an unconventional secretion pathway (137). Like IL-1β, the IL-18 precursor could be cleaved by caspase-8 (138); however, unlike IL-1β, IL-18 is constitutively expressed in myeloid cells and epithelial cells, functions *via* IL-1R5 (IL-18 receptor alpha chain)/IL-1R7 (IL-18 receptor beta chain) ligation, and is antagonized by the IL-18–binding protein (139–141). The predominant cytokine downstream of inflammasome signaling could be either IL-1β (142), IL-18 (143), or a combination of the two (144) in a cell type- and context-dependent manner. IL-18 upregulates the expression of cell adhesion molecules (145), nitric oxide synthesis (146), cytokine and chemokine production (147–149), and IFNγ production in CD4+/CD8+ T cells and NK cells *via* combined action with IL-12 (150). These pro-inflammatory IL-18 effects contribute to the pathogenesis and development of diseases such as COVID-19 (151), inflammatory bowel disease (152), diabetes (153), and cancer (154–156). However, IL-18 also has a protective effect against the progression of age-related macular degeneration (157) and Alzheimer’s disease (158). Therefore, IL-18 downstream inflammasome signaling plays an important role in maintaining homeostasis and inducing inflammation.

Human IL-37, an anti-inflammatory IL-1 family member that has no homolog in mice or chimpanzees, has isoforms (b-e) that contain caspase-1 cleavage sites and enable maturation for increased activity compared to the active precursor without a classical signal peptide (79, 159). Active caspase-1 is required for the secretion of mature IL-37 and the nuclear translocation of intracellular IL-37 in NLRP3 signaling but is not required for the release of its precursors (160). Peritoneal macrophages from transgenic mice expressing native human IL-37 decrease the LPS-induced production of IL-6, IFNγ, TNFα, and IL-1β, along with suppressing the activation of NFκB and MAP kinase as compared to mice harboring caspase-1-uncleavable D20A mutant IL-37, further indicating an unexpected role of caspase-1 in limiting inflammation (161). IL-37 could ameliorate the inflammation in insulin resistance (162), allergic rhinitis (163), and asthma (164–166). However, its expression is upregulated in rheumatoid arthritis (167–169), ankylosing spondylitis (170), Grave’s disease (171), and systemic lupus erythematosus (172–174), with a relatively lower IL-37 level correlating with higher disease activity (175); this indicates the potential anti-inflammatory effect of IL-37 against inflammatory situations.

**Figure 2:**
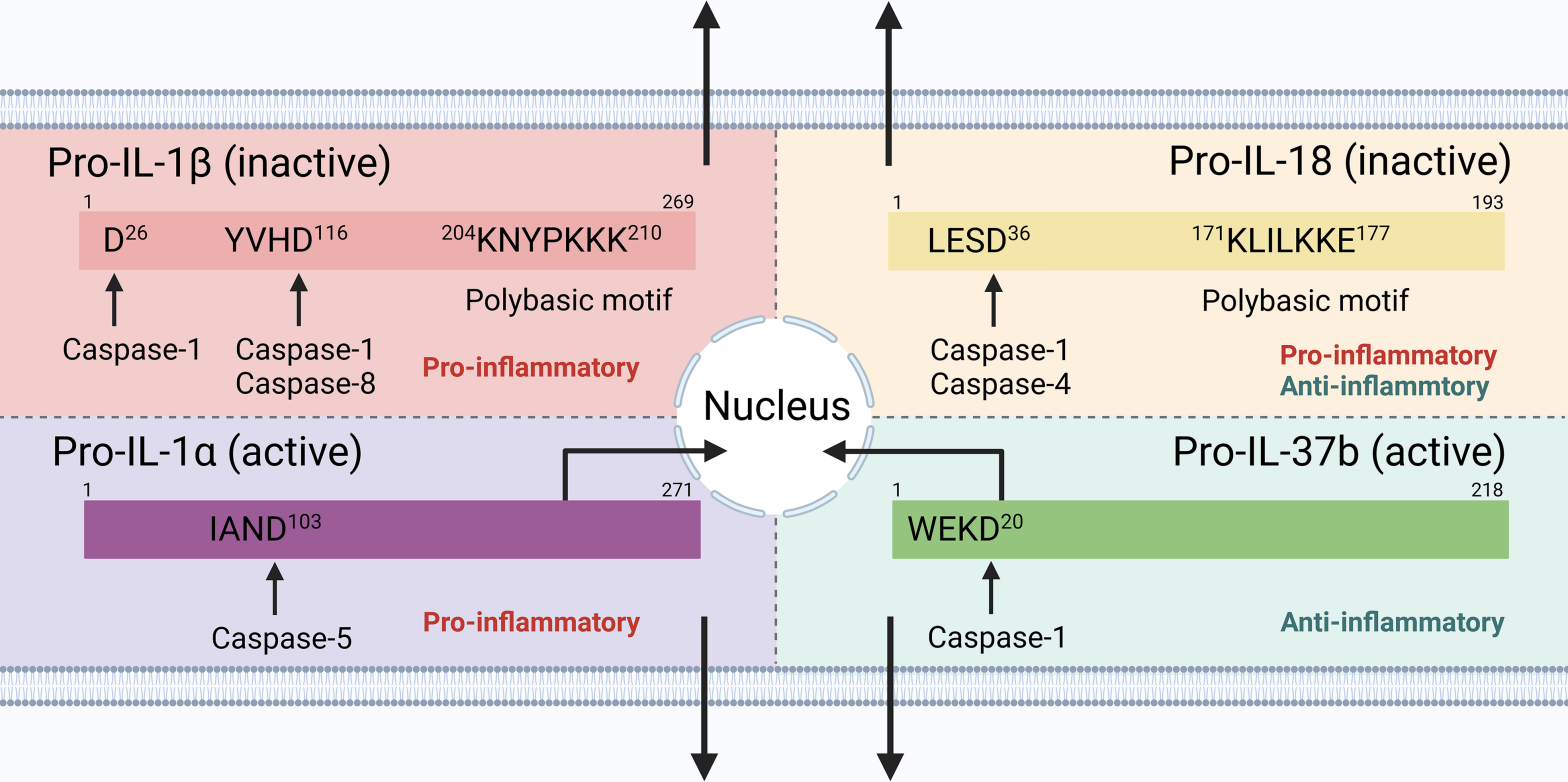
Cytokine processing downstream inflammasome signaling in human cells. IL-1β and IL-18 precursors are inactive and require cleavage by caspase-1 into their mature forms; IL-1α and IL-37 precursors are active, with mature forms that increase the biological activity and receptor affinity. IL-1α and IL-37 could also shuttle to the nucleus to exert their pro-/anti-inflammatory functions.

The maturation and secretion of cytokines downstream of inflammasome signaling, therefore, play an important role in regulating innate and adaptive immunity (**Figure 2**). When considering the extracellular function, cytokine maturation and secretion could be uncoupled from pyroptosis in live and lytic cells. Since IL-1α and IL-37 precursors are active, the role of caspase-1 in pro-IL-1α cleavage could be indirect, and IL-18 may be preferentially produced in non-myeloid cells [e.g., bronchial epithelial cells (143), but PBMCs may act as an exception with large amount of IL-1β production (176)] following inflammasome activation,, this review, therefore, uses IL-1β secretion as a main readout of cytokine maturation and release (if not always), downstream of inflammasome signaling.

## 4 Events upstream of pyroptosis and cytokine secretion in inflammasome signaling

### 4.1 ASC-mediated inflammasome assembly

ASC, encoded by the gene *PYCARD* on chromosome 16p11.2–p12 and first discovered in aggregate form during human HL-60 cells apoptosis, is a 22kDa bipartite adaptor that is composed of a C-terminal caspase recruitment domain (CARD) and an N-terminal pryin domain (PYD) connected with a linker region consisting of 23-amino acids (177). ASC monomers are in their soluble inactive form when resting, and a relatively high thermodynamic energy barrier prevents them from spontaneously oligomerizing into insoluble supramolecular structures (178). However, ASC overexpression may promote oligomerization; thus, special attention is required when interpreting such results (177, 179). In contrast, pathogens (e.g., *Legionella pneumophila*) could target ASC and downregulate its expression to evade effective immune elimination (180). ASC is detected in the spleen, peripheral blood leukocytes, small intestine, lung, thymus, colon, and at very low levels in the brain, heart, and skeletal muscle (181, 182). Intracellular ASC may be present within the cytosol, intracellular compartments (e.g., the Golgi), or the nucleus, as has controversially been observed in different studies owing to discrepancies involving ASC overexpression or the antibodies that are used for ASC detection (183–185). In addition, ASC is constitutively expressed in many immune cells and myeloid cell lines (e.g., THP-1 cells), but not in murine Raw264.7 macrophages (186–188). ASC deficiency may even lead to GSDME-mediated alternative pyroptosis instead of GSDMD-mediated canonical pyroptosis in LPS-primed and ATP-treated Raw264.7 cells, accompanied by the release of pro-IL-1β (189). Several studies have observed “induced ASC expression” in Raw264.7 macrophages, although the exact mechanism remains unclear (190–194).

In addition to versatile functions in inflammasome-independent biological processes (195–200), ASC has established its central role in inflammasome signaling. Briefly, ASC connects the inflammasome sensor and caspase-1 *via* homotypic interactions of CARD-CARD and PYD-PYD, hierarchically organizing and densely packing the ternary inflammasome complex into a micrometer-sized disk-like structure that is denoted a pyroptosome or an ASC speck. ASC specks are usually found in a perinuclear location in myeloid cells but have also been reported in the nuclei of human and zebrafish keratinocytes (179, 201–203). Caspase-8 can also be involved in ASC specks within active or inhibited inflammasomes in a context-dependent manner (189, 204). ASC speck formation have been observed in immune cells such as monocytes in patients with HIV (205, 206), tuberculosis-immune reconstitution inflammatory syndrome (207), severe COVID-19 (208–211), and primary progressive multiple sclerosis (212), neutrophils in patients with sepsis (213), severe COVID-19 (214), and PAMI syndrome (215), CD1c^+^ DCs found in human fibrotic kidney tissue (216), and fibroblasts and CD11c^+^ DCs that are associated with experimental influenza (217). Intracellular ASC speck formation in neutrophils and macrophages can be observed as early as 4 h after Group B *Streptococcus* infection, corresponding to an IL-1β peak in splenic tissue (217). A high proportion (75%) of speck-positive cells undergo pyroptosis, while 76% speck-negative cells in bone-marrow-derived DCs (BMDCs) primed with LPS and treated with nigericin are viable (217). These results highlight the role of ASC specks in events that are downstream of inflammasome signaling.

**Figure 3:**
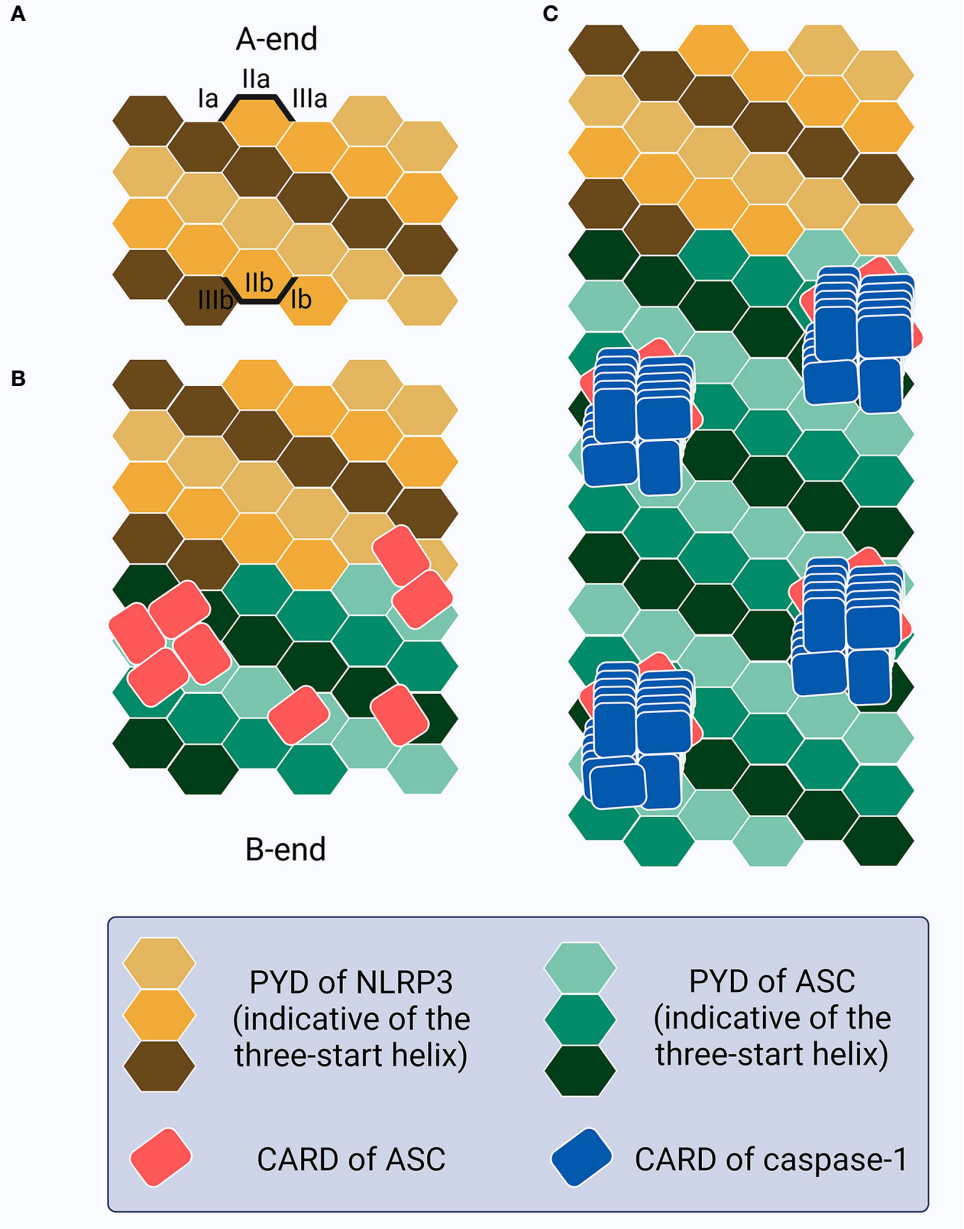
Schematic of the ASC speck formation. **(A)** The PYDs in sensors (e.g., NLRP3) oligomerize as a seed to recruit the PYDs of ASC. **(B)** This homotypic PYD-PYD interaction creates a unidirectional (A-end to B-end), right-handed, three-start helix as a linear ASC filamentous backbone structure with CARDs exposed and self-interacting outside the filaments, resembling buds on the stem surface. **(C)** CARD-CARD interaction between ASC and caspase-1 allows caspase-1 recruitment as a left-handed one-start helix, thus increasing the local concentration and promoting activation.

Notably, ASC^PYD^ and ASC^CARD^ have been proven not to interact with each other in both resting and active cells (218–220), and play different roles in macromolecular assembly (179, 220, 221). The full-length ASC transduction in ASC^-/-^ BMDMs primed with LPS and treated with poly(dA:dT) or ATP triggers ASC speck formation, whereas ASC^PYD^ expression induces only filamentous structures (222). However, ASC^CARD^ expression alone fails to lead to the formation of macromolecular assemblies such as filaments or specks, while full-length ASC with mutant CARD generates specks of larger diameters that are less dense and resemble filaments (222). More precisely, ASC speck formation in inflammasomes that contain PYDs (e.g., NLRP3 or AIM2) requires several steps (181, 222–224) (**Figure 3**): (1) sensor PYDs oligomerize when challenged by PAMPs and/or DAMPs, generating PYD clusters *via* homotypic PYD-PYD interaction within the sensors; (2) PYD clusters act as a seed to recruit the first batch of ASCs *via* sensor^PYD^-ASC^PYD^. More ASC is subsequently recruited *via* ASC^PYD^-ASC^PYD^ interaction with six adjacent counterparts, through charge-based asymmetric interface-types I (Ia-Ib, the largest and conserved, intrastrand), II (IIa-IIb, interstrand), and III (IIIa-IIIb, interstrand), creating a right-handed, three-start helix. The linear ASC filamentous backbone structure is thus unidirectionally and exclusively elongated and the CARDs are exposed to self-interact outside the filaments; (3) ASC^CARD^-ASC^CARD^ interaction, both within the same and across different filaments, forms seeds for recruitment of the first batches of caspase-1 *via* ASC^CARD^-caspase-1^CARD^, after which more caspase-1 is recruited *via* caspase-1^CARD^-caspase-1^CARD^. ASC filaments are thus cross-linked and condensed into well-packed ASC specks and the concentration of monomer caspase-1 increases locally, facilitating its activation. Caspase-1 is over stoichiometric (approximately 3.5-fold) to ASC in the inflammasome complex, suggesting that there needs to be sufficient free ASC^CARD^ left following ASC^CARD^-ASC^CARD^ interaction for caspase-1 binding if effective activation is to occur (179). In terms of inflammasome sensors that contain CARDs (e.g., NLRC4, but not human CARD8), although caspase-1 can be recruited directly through sensor^CARD^-caspase-1^CARD^ interaction (except for human NLPR1), ASC^CARD^ may also interact with sensor^CARD^ to form a bridge, followed by the recruitment of other ASCs to form filaments through ASC^PYD^-ASC^PYD^, and specks *via* ASC^CARD^-ASC^CARD^, interaction (181). Notably, chloride-free or efflux conditions could lead to ASC oligomerization and the formation of inactive specks that are incapable of activating caspase-1-mediated LDH release and IL-1β secretion until a concomitant potassium efflux is added to the NEK7-dependent NLRP3 oligomerization (225, 226). The chloride-dependent ASC oligomerization or inactive speck formation may act as a dynamic, reversible step in the formation of competent ASC specks in inflammasome signaling, with further information required for activation. Once all the triggers are ready and primed for action, the formation of the ASC speck exhibits a prion-like effect (227–229). As soon as the sensor PYDs form clustering seeds from which the conformational changes lower the energy barrier associated with ASC oligomerization, ASC is homogenously recruited from its initial location following an energetic gradient until all free ASCs are depleted (230). This leads to an all-or-none cascade at the single-cell level; the inflammasome complex is activated when ASC speck formation succeeds and is not activated if ASC specks formation fails. Thus, as an adaptor, bridge, and amplifier, ASC functions as a “central” hub in inflammasome signaling, transducing signals from the upstream stimuli-provoked-sensors to downstream caspase-1 to ultimately facilitate pyroptosis and cytokine secretion. Intracellular ASC specks have also been reported to co-aggregate stably with antigenic proteins, indicating a role for antigen presentation in shaping adaptive immunity against intracellular infection (231).

Since ASC is the central hub in inflammasome signaling, its function is tightly controlled, including in the sensor level (232), and also in its interactions with other ASC molecules and the sensor/caspase-1 by alternative splicing (233–235), post-translational modifications (236–241), PYD-only proteins (242–244), and CARD-only proteins (245–247). ASC oligomerization and speck formation can be disrupted by gene mutation (248, 249), pathogens (250), bioactive extracts (251–254)/derivatives (255–257)/metabolites (258), cytokines (259), complements (260), or chemicals (261–263) or further stabilized by L-plastin (264), inhibiting or facilitating downstream signaling, respectively. Additionally, ASC specks are detected in the extracellular environment (229, 265) in pathological conditions such as brain injury (266, 267), recurrent ischemic stroke (268), and Alzheimer’s disease (269, 270). These extracellular ASC specks may activate extracellular caspase-1 that has been released from provoked cells or intracellular caspase-1 in other recipient cells (71, 271, 272) following the internalization and release of specks from endosomes, facilitating IL-1β maturation and/or GSDMD processing (228, 273), or nucleate other proteins such as Amyloid-β (274–276) for disease progression. A recent study also provides evidence of the role of post-pyroptotic extracellular ASC specks in MSU induced-gouty arthritis and antigen-induced arthritis, in which nanobodies against mouse ASC could ameliorates inflammation (71). This may indicate fine-tuning of the cell-extracellular milieu-cell alarm transduction against threats in which ASC can act as a central adaptor, bridge, and amplifier on a cellular level, or led to unrestrained inflammation and dissemination, if dysregulated. The intracellular and extracellular pro-inflammatory roles of ASC specks also provide potential targets for developing therapeutic drugs. Further studies are required for a better understanding of the dynamics and functions of intracellular and extracellular ASC specks.

Collectively, ASC speck formation, including the generation of filaments by ASC^PYD^ and condensation by ASC^CARD^, provides multiple potential sites for the activation of caspase-1, thereby serving as amplification machinery for inflammasome signal transduction. Once the inflammatory “threshold” is reached under physiological or pathological conditions, the speck is formed as an all-or-none prionoid event or an on-or-off response that needs to be carefully controlled by the host immune system. However, the different requirements of ASC and specks can exist in inflammasome sensors containing PYDs and CARDs, or even in the same inflammasomes with different mutations, and the downstream events may therefore become uncoupled (discussed later). The exact mechanisms of ASC, especially the caspase-1-activation-driven functions, in inflammasome signaling, therefore, warrant further investigation.

### 4.2 Caspase-1 dynamics driven by inflammasome activation

Caspase-1, a cysteine protease encoded by *CASP1* on chromosome 11q22.3, is known as the main executor caspase in canonical inflammasome signaling (277–280). Unlike the apoptotic caspases that can be classified into initiators and executioners, caspase-1 can act as both initiator and executioner, thus contributing to the inflammatory response in a versatile manner as well as affecting other biological processes such as lipid metabolism and the cell cycle (281–284). Strikingly, although caspase-1 can exert an anti-inflammatory effect by processing IL-37 [leading to the downregulation of IL-6 activity (160) or impairment of NLRP3 function (285)] or by inactivating IL-33 [resulting in the reduction of T-helper type 2 immunity (286)], caspase-1 is commonly regarded as a potent pro-inflammatory regulator that directly cleaves GSDMD, pro-IL-1β, and pro-IL-18, promoting pyroptosis and cytokin secretion. Caspase-1 mutations may lead to inefficient auto-processing and reduced catalytic activity, abolishing downstream signaling, disrupting the equilibrium of caspase-1-centered inflammatory regulation, and resulting in numerous inflammatory disorders (e.g., periodic fever syndromes) (287–289). A better understanding of caspase-1 dynamic machinery is fundamental if the mechanisms of the associated diseases are to be deciphered.

**Figure 4:**
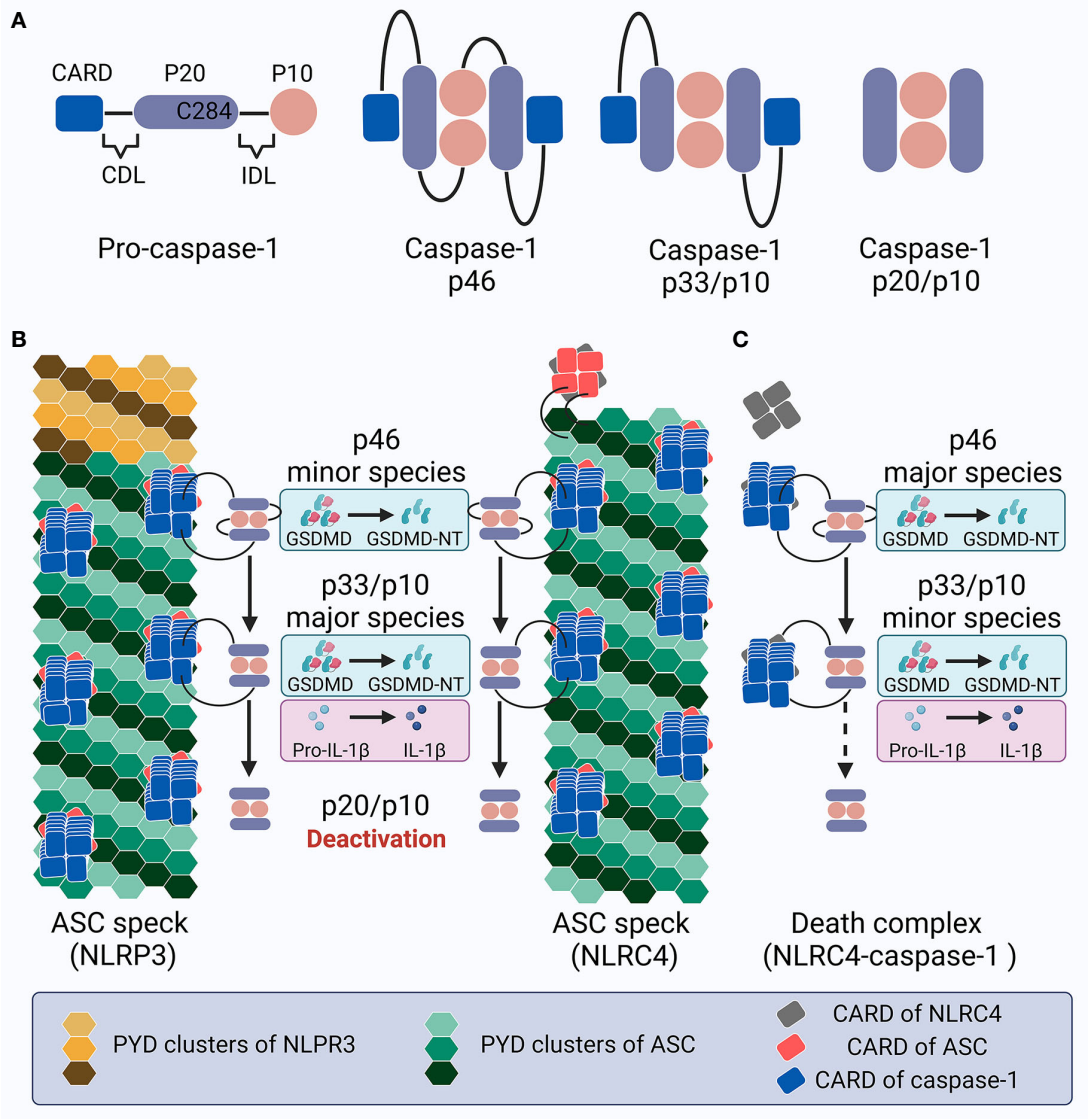
Schematic showing caspase-1 activation and deactivation. **(A)** Structure of pro-caspase-1 and caspase-1 species generated by dimerization and auto-processing during inflammasome activation. **(B)** Upon NLRP3 or NLRC4 inflammasome activation in cells expressing ASC, specks recruit pro-caspase-1 molecules, leading to proximity-induced dimerization, generation of p46 species (minor), and later auto-proteolysis; this process first occurs at the IDL to generate p33/p10 species (major), and subsequently at the CDL to create and dislocate the inactive p20/10 species. The p46 species cleaves GSDMD into GSDMD-NT, while p33/p10 species cleaves both GSDMD and IL-1β. In contrast, the p20/p10 species is incompetent for processing GSDMD and IL-1β. Within the ASC speck, caspase-1 activation and deactivation is rapidly promoted with a high turnover. **(C)** Upon NLRC4 inflammasome activation in ASC deficient cells, the turnover of p46 into p33/p10, and especially p33/p10 into p20/p10 (illustrated with a dotted arrow), slows down; this leads to prolonged caspase-1 activity, with p46 and p33/p10 as the major and minor species, respectively, which may contribute to the pyroptosis-predominant uncoupling without massive IL-1β release. This inflammasome complex is called the death complex.

Caspase-1 is composed of an N-terminal CARD, a CARD domain linker (CDL), the large subunit p20 that contains catalytic residues (C285 and H237 in human/C284 and H236 in mouse), an inter-domain linker (IDL), and the small subunit p10 that is involved in dimerization (277, 290). Both CDL and IDL are sensitive to proteolysis to generate different species (**Figure 4A**). While alternative splicing, posttranslational modification, and proteins associated with recruitment may lead to complexity in caspase-1 activity variation (291–294), here we mainly focus on the mechanisms of caspase-1 dynamics within the inflammasome platform for processing GSDMD and IL-1β. Pro-caspase-1 is present as zymogen monomers without catalytic activity in its resting state (80). Akin to ASC, caspase-1 monomers are prone to oligomerization under high concentrations in certain experimental conditions such as overexpression, which may not reflect the natural dynamics in physiological cellular conditions (295). Numerous studies have shown that full-length pro-caspase-1 is activated by proximity-induced dimerization followed by auto-proteolysis when recruited by inflammasomes; this first occurs at the IDL and subsequently at the CDL, generating an active p20/p10 tetramer (namely the p20_2_/p10_2_ heterotetramer) as the mature caspase-1 to cleave GSDMD and IL-1β (83, 296–300). However, *in vitro* study has reported that active caspase-1 is unstable and short-lived (half-life = 9 min) at an initial concentration of 10 nM at 37°C, and its promiscuity towards multiple natural substrates increases when its concentration exceeds a threshold of ~50–100 nM (301). Hence, caspase-1 activity under cellular conditions exhibits better specificity towards limited substrates (302, 303). Moreover, endogenous caspase-1 activity towards its preferred substrate and the amount of active caspase-1 captured by the activity probe decrease sharply despite the accumulation of processed caspase-1 (301). These results suggest tightly-organized activation and deactivation of caspase-1. A rapid and drastic loss of quaternary structure (e.g., losing the p10 subunit by auto-processing at the high caspase-1 concentration of 2 μm) may contribute to the instability of the p20/p10 tetramer, leading to caspase-1 deactivation (297, 304). However, it is debatable whether such a high concentration would occur in the physiological environment. Therefore, although the hypothesis that the p20/p10 tetramer is the active form of caspase-1 has gained popularity over the past few years, the exact machinery and mechanism of caspase-1 dynamics within the inflammasome complex, and especially its activation and deactivation, remain enigmatic.

One recent study indicated that the biotin-VAD-fmk probe, which binds covalently to active caspase-1, interacts predominantly with the p33/p10 species (processed at IDL but not CDL) and to a lesser extent the p46 full-length caspase-1 dimers in LPS-primed nigericin-stimulated BMDMs, indicating that these two species comprise the main active forms under intracellular inflammasome activation (305). The dimeric full-length p46 can effectively process GSDMD but not IL-1β, while p33/p10 can cleave both GSDMD and IL-1β (305). The p33/p10 dimers were observed to retain a relatively stable rate of activity over time before being cleaved at the CDL to form the p20/p10 tetramer, suggesting that the p20/p10 tetramer, which is generally regarded as “mature caspase-1”, is an inactive form under physiological cellular circumstances, although the recombinant p20/p10 tetramer exhibits high activity on the caspase-1 substrate YVAD *in vitro* (305). The loss of p20/p10 tetramer catalysis on downstream substrates under cellular circumstances might be due to: (1) a decrease in the local concentration when the CDL in p33/p10 is cleaved that leads to the subsequent dissemination of p20/p10 from the inflammasome complex, which may allow the caspase-1 concentration to be maintained *via* binding, and (2) the quaternary structure of caspase-1 dimers remains stable only when recruited to the inflammasome hub; the p20/p10 tetramer is released and when the CDL is cleaved, leading to instability (which is also partly due to its decreased concentration). Therefore, the caspase-1 dynamics program is thought to include several steps (305) (**Figure 4B**): (1) full-length pro-caspase-1 monomers are recruited to the inflammasome sensor with the help of ASC; (2) proximity-induced dimerization produces full-length p46 dimers in the form of partially active caspase-1, with the ability to process GSDMD but not IL-1β; (3) the autoproteolysis of p46 caspase-1 dimers at IDL generates p33/p10 dimers in the form of fully active caspase-1, with the ability to process both GSDMD and IL-1β; and (4) the autoproteolysis of p33/p10 dimers at CDL creates p20/p10 tetramers, which are later released from the inflammasome complex due to the destroyed CARD-CARD interaction. Since the disassociated p20/p10 tetramer is not stable and its cellular concentration is lowered along with its dissipation, it is no longer able to efficiently process GSDMD and IL-1β (301) and the activated caspase-1 is deactivated. Hence, the inflammasome platform possesses the capacity to both activate and deactivate caspase-1, acting as a holoenzyme that comprises an elegantly self-limiting system with feedback loops and regulating the intensity and duration of caspase-1 activity and thereby the downstream processing of GSDMD and IL-1β.

This caspase-1 dynamic program is tightly regulated by inflammasome size and is associated with caspase-1 recruiting sites and the cell type specifying the expression levels of relative molecules. Inflammasomes with a larger number of caspase-1 recruiting sites (e.g., large sensor-ASC-caspase-1 complexes) may generate p20/p10 more effectively, thereby contributing to higher caspase-1 turnover. In contrast, the NLRC4 inflammasomes in ASC^-/-^ macrophages (small NLRC4-caspase-1 complexes) as compared to those in WT cells (large NLRC4-ASC-caspase-1 complexes) or inadequately-organized specks (medium NLRP3-ASC^+/-^-caspase-1 complexes) as compared to well-organized ASC specks (large NLRP3-ASC-caspase-1 complex) leads to accumulation of the p46 and p33/p10 dimers, prolonging the duration of caspase-1 activity (**Figure 4C**) (305). Moreover, the number of caspase-1 recruiting sites in inflammasomes can negatively influence the p46-to-p33/p10 dynamics, as evidenced by the higher amount of active p46 than p33/10 that has been observed in the NLRC4-caspase-1 inflammasome (305, 306). Therefore, the inflammasome complex is responsible for regulating caspase-1 activity, at least in the intracellular environment. Notably, the p20/p10 caspase-1, with no effective activity under cellular conditions, may still be regarded as evidence for the previous presence of the p46 and p33/p10 species, indicating full caspase-1 maturation. In addition, as caspase-1/GSDMD-mediated pyroptosis and cell lysis may lead to the extracellular release of inflammasome components (e.g., ASC specks, full-length/cleaved caspase-1), whether this timer of activation and deactivation also functions in this way in extracellular space remains unknown (83, 307). It has been reported that p20 caspase-1 that is induced by NLRP3 inflammasome activation in THP1 cells and released to extracellular supernatant exhibits two forms: a 60 kDa protein consistent with the p20/p10 tetramer, and a high molecular fraction (≥200 kDa) with fluorogenic substrate activity (308). The authors hypothesize that this high molecular fraction containing p20 caspase-1 may help extend the duration of active caspase-1. Further investigation is required to elucidate the underlying mechanisms of this undefined extracellular complex and inflammasome timer.

Being CARD-containing inflammasomes, mouse Nlrp1b, human NLRP1, and CARD8 inflammasomes seem to present somewhat different caspase-1 dynamics from that in NLRC4. Nlrp1b consists of CARD, function-to-find domain (FIIND), LRR, NACHT, and a cleavage site for lethal factor near its N-terminus (10). In Raw264.7 cells that are generated to stably express ASC. ASC speck formation and caspase-1 cleavage have been observed to show Nlrp1b signaling in response to anthrax lethal toxin (309). However, unprocessed caspase-1 is sufficient to induce IL-1β and LDH release in the absence of ASC in mouse BMDMs and MBDCs treated with anthrax lethal toxin (310, 311). The expression of auto-cleavage mutant C71 (D103N, D122N, D296N, D308N, D313N, and D314N), but not catalytic dead caspase-1, was found to restore the inhibited IL-1β and LDH release in caspase-1^-/-^ cells in response to anthrax lethal toxin, but not to nigericin (311). Caspase-1 dimer also matures IL-1β in genetically engineered mouse embryonic fibroblasts without inflammasome activators (312). These data may suggest the dual competent roles of the full-length caspase-1 dimers in both processing IL-1β and inducing pyroptosis independently of ASC specks, although another study reports a necessary role of IDL cleavage of caspase-1 for GSDMD processing following Nlrp1b activation (313). Compared to the mouse Nlrp1b, human NLRP1 and CARD8 also contain the FIIND and the CARD; however, the former possesses an N-terminal PYD (reported as dispensable or autoinhibitory in inflammasome activation) while the latter has no NACHT and LRR (314). Unlike mouse Nlrp1b, human Nlrp1b and CARD8 inflammasomes are not activated by anthrax lethal toxin due to the lack of cleavage site for lethal factor. Similar in the NLRP1b inflammasome (315), “Pro-caspase-1-dependent pyroptosis” is also found in CARD8 inflammasome signaling in response to the DPP8/9 inhibitor Val-boroPro (316, 317); however, pro-caspase-1 processing is hypothesized to be observed if the cleavage products are not readily released into the supernatant. A recent study shows that when exposed to Val-boroPro, human NLRP1 requires ASC to mediate GSDMD processing without NLRP1^CARD^-caspase-1^CARD^ interaction, while CARD8 inflammasome processes GSDMD in an ASC-independent manner without CARD8^CARD^-ASC^CARD^ interaction and speck formation (313). Notably, the ASC-independent pyroptosis may require IDL cleavage of caspase-1 in the CARD8 inflammasome (313). These data may suggest the enigmatic complexity of caspase-1 dynamics mechanisms in CARD-containing inflammasomes other than NLRC4.

Additionally, murine caspase-11 directly senses the intracellular LPS for non-canonical inflammasome activation and GSDMD-mediated pyroptosis, which may lead to non-canonical NLRP3 inflammasome activation and caspase-1-dependent IL-1β and IL-18 release, contributing to bacterial clearance and host survival in a cell type-dependent manner (318–320). Human caspase-4 and caspase-5 function in a similar way to caspase-11 in inducing pyroptosis; however, caspase-4 is also reported to cleave pro-IL-18 (321–323). Upon LPS binding, caspase-11 undergoes oligomerization and proximity-induced activation (324). Like caspase-1, dimerization renders caspase-11 the basal proteolytic activity, while the auto-cleavage at CDL and IDL convey varied activity, and the fully active p32/p10 species is generated following cleavage at D285 within the IDL (325). In this way, caspase-11 is activated *via* functional crosstalk with canonical inflammasome signaling under various conditions (320). Notably, as caspase-1^-/-^ mice usually have mutations in the caspase-11 gene because of the chromosomal proximity of these two genes in the mouse genome, careful consideration should be given to the interpretation of results using “caspase-1 KO mice” which are deficient in both caspase-1 and caspase-11 (326). Despite these classic canonical caspases, as mentioned above, the traditional apoptotic caspase-8 may also play a key role in mediating IL-1β maturation and GSDMD processing with complex crosstalk among apoptosis, necroptosis, and pyroptosis (327–329). Caspase-8 could act as both an effector and a regulator in NLRP3 inflammasome activation, or as a backup when caspase-1 is absent (330–332). The catalytically inactive caspase-8 mouse model A (Casp8^C362mut^) with both individual and combined RIPK3, MLKL, caspase-1, or ASC deficiencies, may shed light on how caspase-8 participates in regulating inflammation and homeostasis to decide the fate of cell and host (329).

Collectively, three modes of caspase-1 dynamics in inflammasome activation have been outlined above: (1) the p20/p10 tetramer that are previously and generally accepted as active caspase-1; (2) p46 (for GSDMD processing)-p33/p10 (for both GSDMD and IL-1β processing)-p20/p10 (deactivated species) caspase-1 dynamics in PYD-containing and NLRC4 inflammasomes; (3) complex caspase-1 dynamics in mouse Nlrp1b, human NLRP1, and CARD8. As the activation mechanism of caspase-1 and the exact structure of active caspase-1 remain to be fully elucidated, we would like to discuss the caspase-1 forms (e.g., p20 caspase-1) obtained in different studies to confirm caspase-1 activity in the following section.

## 5 Uncoupling events downstream of inflammasome signaling

### 5.1 Cytokine-predominant uncoupling: Hyperactivation

Cytokines such as TNF-α and IL-6 may be produced and secreted in response to TLR stimuli in activated cells; in contrast, IL-1β release requires expression, maturation, and secretion in pyroptotic and hyperactive cells, with the former amplifying robust IL-1β release in a short time window before dying and the latter remaining viable to add IL-1β into the cytokine reservoir, prolonging inflammation and leading to cytokine-predominant uncoupling downstream of inflammasome signaling (55).

Hyperactivation can be observed in both immune and nonimmune cells in response to DAMPs. Extracellular ATP, a common pyroptosis inducer for macrophages, triggers IL-1β secretion without LDH release in human PBMCs and THP1 cells that have been primed with LPS (333–335). oxPAPC, which refers to oxidized phosphorylcholine derivatives found in dying cells and damaged tissues, induces IL-1β secretion in LPS-primed DCs (GMCSF-DCs) but not macrophages in NLRP3/ASC/caspase-1/caspase-11-dependent manner without the release of LDH and damage to the functional mitochondria and cell membrane integrity (336). Co-immunization with LPS and oxPAPC also enhances CD4+ T cell activation in mice by promoting the secretion of IL-2, IL-17, and IFN-γ as compared to LPS or oxPAPC alone, indicating the role of hyperactive DCs in promoting T cell–mediated immunity (336). The authors further reported that oxPAPC or its components (e.g., PGPC) bind to CD14 on the cell membrane in LPS-primed DCs and macrophages, promoting endocytosis with prolonged IL-1β and IL-18 secretion as compared to ATP-treated macrophages *in vitro*, enhancing inflammation but not lethality in mouse sepsis (337). This group also proved that hyperactive FLT3L-DCs increase their capacity to migrate to skin-draining lymph nodes in an inflammasome-independent manner compared to their naive or active counterparts and sustain their ability for prolonged IL-1β release in an inflammasome-dependent manner (338). Notably, hyperactive DCs promote the strongest CD8+ T cell generation and effector responses among their pyroptotic and active counterparts in an inflammasome-dependent manner (338), and hyperactive stimuli (LPS+oxPAPC or PGPC) increase the rate and magnitude of effector and memory T cell generation in mouse models, contributing to long-term anti-tumor immunity (338). Another endogenous DAMP, the membrane attack complex (MAC), which is formed by C5b-C9 as a result of complement-system activation, also triggers sub-lytic hyperactivation. MAC internalization into EEA1^+^ endosomes by LPS-primed THP1 cells and human monocyte-derived macrophages induces NLRP3 inflammasome activation and IL-1β secretion in the absence of LDH release (260). Complement-system activators also induce IL-1β secretion in viable LPS-primed mouse BMDMs in an NLRP3/ASC/caspase-1-dependent and NLRC4-independent manner (339). Additionally, amyloid-β could induce NLRP3/ASC/caspase-1-mediated IL-1β release in live LPS-primed microglia (270). Together, these DAMP-triggered hyperactive cells may contribute to prolonged inflammation *via* consistent IL-1β secretion.

Hyperactivation is also detected in immune and nonimmune cells under pathogen infection. Low doses of nigericin (0.5 μM) may induce sustained IL-1β secretion with minimal cell death in a LPS-primed immortalized mouse BMDMs (iBMDMs), whereas high doses (20 μM) lead to IL-1β secretion (albeit less in total) and massive LDH release (53); in contrast, the cytotoxic effect of nigericin treatment (20 μM for 30 min) on LDH release in LPS-primed monocytes (monocytes from human PBMCs versus THP1 cells) is controversial with regards to LDH secretion (335, 340). Regardless of the second signal, LPS alone is sufficient to induce IL-β secretion in human monocytes without LDH release (341–344). This alternative inflammasome activation is dependent on the TLR4-TRIF-RIPK1-FADD-CASP8 axis upstream of NLRP3 and independent of K+ efflux and ASC speck formation (343). This mode is quite different from classical NLRP3 signaling, which requires two signals—the first for priming (pro-IL-1β transcription and translation) and the second for licensing (IL-1β maturation)—and LPS or ATP treatment alone is insufficient to initiate the NLRP3-mediated release of IL-1β from macrophages (68, 84). Caspase-8 cleaves neither NLRP3 nor IL-1β in this situation and does not function upstream of canonical and non-canonical NLRP3 activation in murine macrophages; therefore, a unique role is suggested for caspse-8 in association with alternative inflammasome signaling in human monocytes (343, 345). Another study found that caspase-4/-5 are also required in IL-1β secretion in live LPS-treated human monocytes, where LPS-TLR4 binding leads to LPS internalization and cytosolic localization (346). Despite LPS, other bacteria or bacterial products may also trigger hyperactivation. The gram-negative *Salmonella enterica serovar Typhimurium* infection induces IL-1β secretion in live human primary monocytes regardless of LPS priming, whereas the NLRP3 inhibitor MCC950 and extracellular KCL dampen IL-1β release (344). PNG from OatA-deficient *Staphylococcus aureus* or its lysosomal degradation product NAG triggers NLRP3 activation and IL-1β secretion in LPS-primed human and mouse macrophages and DCs by promoting hexokinase dissociation and mtDNA release from mitochondria into the cytosol without the collapse of total mitochondrial function (347). LDH release is not detected in these cells, and no K+ efflux is required (347). The crosstalk between glycolysis and inflammasome signaling resulting from pathogen PNG detection and degradation in phagocytes may increase neutrophil infiltration *in vivo* (347). Another study revealed that the OatA-deficient *S. aureus* or oxPAPC components induce ASC speck formation and IL-1β secretion through GSDMD pores in live iBMDMs and mouse BMDMs with intact mitochondrial and phagocytic activity (348). GSDMD deficiency inhibits IL-1β secretion but not pro-IL-1β processing (348). Moreover, the percentage of PI^+^ cells, which may indicate the extent of GSDMD pore formation, is less in iBMDMs treated with OatA-deficient *S. aureus* or oxPAPC components than in those treated with nigericin, which may explain why the former two populations are hyperactivated rather than killed (348). Among the hyperactive stimuli, the more cytotoxic PGN induces less IL-1β secretion, while the weaker cytotoxic OatA-deficient *S. aureus* triggers more IL-1β release (348). Membrane repair mechanisms (e.g., ESCRT) may also contribute to the sublytic GSDMD pore formation (26); however, whether and how exactly the ESCRT-III machinery functions in hyperactivation remain to be further defined as its inhibition both increase pyroptosis and IL-1β release (63, 349). These data may suggest the complex regulation of GSDMD pore formation in association with unbalanced IL-1β release and cell death, considering the different inflammation levels and cell life span associated with pyroptosis.

Additionally, GSDMD, but not pore formation, may also act as the key regulator in hyperactivation in some scenarios. Intracytosolic *Listeria monocytogenes* or cytosolic LTA induces IL-1β and IL-18 secretion in mouse BMDMs in a NLRP6/ASC/caspase-11/caspase-1-dependent manner without detectable LDH release or GSDMD cleavage (350). The exact mechanism by which IL-1β and IL-18 are released in this situation remains elusive, as these mature cytokines are also significantly observed within the cell lysates of LTA-transfected BMDMs versus those with LPS (350). GSDMD is also not cleaved in young adult mice colonic (YAMC) epithelial cells that are primed with LPS and treated with ATP; however, polyubiquitinated pro-IL-1β and its mature form, GSDMD, full-length caspase-8, and its p18 form are released into the supernatant in WT and Gsdmd^D276A^ (resistant to cleavage) cells without PI intake (75). This non-pyroptotic secretion of IL-1β is mediated by the GSDMD-dependent release of extracellular vesicles (EVs), which contain inflammasome components and the E3 ligase NEDD4, Hsp90 cochaperone Cdc37, ESCRT, and other proteins (75). In these ways, a hyperactive-like state is achieved without GSDMD pores; however, GSDMD is still involved.

The hyperactive-like state can also be observed in neutrophils (351). Neutrophils primed with LPS and infected with *S. Typhimurium* exhibit robust and prolonged IL-1β secretion in a NLRC4/caspase-1-dependent manner without significant LDH release, and ASC deficiency inhibits optimal IL-1β secretion (142). GSDMD deficiency decreases IL-1β release in LPS-primed neutrophils 1 h after *S. Typhimurium* infection; however, comparable IL-1β release is induced 3 h post-infection, along with slightly elevated LDH release, indicating that the late stage of IL-1β secretion is independent of sublytic GSDMD pore formation and implying the possible initiation of other cell death pathways such as apoptosis in GSDMD KO settings (90). Similar uncoupling events are also observed in DNA/poly(dA:dT)-induced AIM2 and nigericin/ATP-induced NLRP3 signaling in neutrophils that are resistant to caspase-1/GSDMD-mediated pyroptosis (39, 52, 68, 142, 352–355). However, the exact mechanism by which IL-1β is released in pyroptosis-resistant neutrophils remains unclear, as cleaved GSDMD could be observed in azurophilic granules and autophagosomes but not in the neutrophil plasma membrane, while maximal IL-1β release requires GSDMD (352). In contrast to these caspase-1 activating stimuli, cytoplasmic LPS triggers coupling events in which caspase-11-/GSDMD-dependent LDH is released and noncanonical NLRP3-dependent IL-1β is secreted together with noncanonical neutrophil extracellular trap formation (NETosis) against cytosolic infection (39).

Despite the GSDMD-dependent pathways, the intrinsic mechanism by which cells become hyperactive is poorly understood. Recently, the role of SARM, the sterile α and heat armadillo motif-containing protein, in regulating the switch between hyperactive and pyroptotic states has been established (356). SARM deficiency does not regulate the mRNA or protein expression of NLRP3, ASC, and pro-IL-1β; however, it enhances IL-1β processing and secretion and decreases LDH release in BMDMs and iBMDMs primed with LPS or Pam_3_CSK_4_ and treated with nigericin, while rescue experiments restore LDH release and reduce IL-1β secretion to similar levels to those observed in their WT counterparts (356). Interestingly, p20 caspase-1 expression, ASC speck oligomerization, and GSDMD cleavage are significantly upregulated in Sarm^-/-^ cells. Further, SARM clusters can lead to mitochondrial depolarization, which may facilitate pyroptosis, and interact with NLRP3, interfering with NLRP3-ASC interaction *via* its TIR domain and decreasing p20 caspase-1 levels in canonical and non-canonical NLRP3 signaling (356). In line with this process, the hyperactivating stimulus PGN causes no SARM clustering and mitochondrial depolarization in LPS-primed BMDMs, suggesting a possible cell-intrinsic mechanism for SARM and the mitochondrial metabolism in balancing hyperactivation and pyroptosis (356). Another study shows that neutrophils express negligible SARM protein compared with macrophages, and SARM transfection decreases the IL-1β secretion in neutrophils that have been pretreated with ATP before treatment with LPS+ATP while showing no effect on LDH release in untreated cells (355). In contrast, another study reported that four independent Sarm^-/-^ BMDM cell lines exhibit comparable LDH release and IL-1 secretion with WT cells (357). The exact role of SARM in cell hyperactivation thus requires further investigation.

Collectively, hyperactivation can be observed in immune and nonimmune cells downstream of multiple inflammasome signaling (**Table 1**). In this way, IL-1β may act as a potent pro-inflammatory regulator in organizing threat elimination and host defense in live cells. Additionally, the data indicate at least two differential roles for GSDMD under hyperactivation: 1) sublytic pore formation on the cell membrane; 2) GSDMD-dependent non-lytic promotion (75, 352). Further study is required to clarify the GSDMD-dependent and independent mechanisms in regulating hyperactivation.

**Table 1:** Hyperactive cells secreting IL-1β without LDH release downstream of NLR inflammasome signaling.

Cell	Stimuli for signal 1	Stimuli for signal 2	ASC	ASC speck	Caspase-1	p20 or p10 caspase-1	Caspase-11/-4/-5	Caspase-8	NLRP3	K^+^ efflux	LDH	GSDMD	Damage of mitochondria	IL-1β	Reference
PMA differentiated THP1 cells	LPS	ATP	NA	NA	NA	NA	NA	NA	NA	NA	NO	NA	NA	YES	(333)
Human PBMCs	LPS	ATP	NA	NA	NA	NA	NA	NA	NA	NA	NO	NA	NA	YES	(334)
Human PBMCs	LPS	ATP	NA	NA	YES	YES (inhibitor assay)	NA	NA	NA	NA	NO	NA	NA	YES	(335)
Primary DCs differentiated from mouse bone marrow	LPS	oxPAPC	YES	YES	YES	NA	YES/NA/NA	NA	YES	NO	NO	NA	NO	YES	(336)
Primary DCs from mouse bone marrow	Pam_3_CSK_4_	oxPAPC	NA	NA	YES	NA	NA	NA	NA	NA	NO	YES	NA	YES	(52)
Primary DCs differentiated from mouse bone marrow	LPS	PGPC or POVPC	NA	NA	YES	NA	YES/NA/NA	NA	YES	NA	NO	NA	NA	YES	(337)
DCs differentiated from mouse bone marrow using Fms-like tyrosine kinase 3 ligand (FLT3L)	LPS	PGPC	YES	YES	YES	NA	YES/NA/NA	NA	YES	NA	NO	NA	NA	YES	(338)
PMA differentiated THP1 cells and human monocyte-derived macrophages	LPS	Membrane attack complex (MAC)	YES	YES	YES	YES (activity assay)	NA	NA	YES	NA	NO	YES	NA	YES	(260)
Mouse BMDMs	LPS	Serum-opsonized zymosan, *Leishmania major*, and inulin	YES	YES	YES	YES (FAM-FLICA™ Caspase-1 assay)	NA	NA	YES	YES	NA (but no increase in % of annexin 7-AAD^+^ cells)	NA	NA	YES	(339)
Mouse primary microglia	LPS	Soluble Aβ oligomers and protofibrils	YES	YES	YES	YES	NA	NA	YES	NA	NO	NA	NA	YES	(270)
Human primary monocytes isolated from PBMCs and BLaER1 cells	LPS	NO	YES	NO	YES	YES	NA	YES	YES	NO	NO	NA	NA	YES	(343)
Human primary monocytes isolated from PBMCs	LPS	NO	NA	NA	YES	YES	NA/YES/YES	NA	YES	NA	NO	NA	NA	YES	(346)
Primary human monocytes isolated from PBMCs	LPS	NO	NA	NA	NA	NA	NA	NA	YES	NA	NO	NA	NA	YES	(344)
Primary human monocytes isolated from PBMCs	LPS (not necessary)	*S. typhimurium*	NA	NA	NA	NA	NA	NA	YES	YES	NO	NA	NA	YES	(344)
Mouse BMDMs and dendritic cells, human macrophages, and dendritic cells	LPS	PNG or its lysosomal degradation product NAG (intracellular) form OatA-deficient *S. aureus*	NA	NA	YES	YES	NA	NA	YES	NO	NO	NA	NO	YES	(347)
iBMDMs and mouse BMDMs	LPS	OatA-deficient *S. aureus*, PGPC or POVPC	YES	YES	NA	NA	NA	NA	NA	NA	NO	YES	NO	YES	(348)
Mouse BMDMs	NO	*Listeria monocytogenes*	YES	NA (but in iBMDMs, YES)	YES	YES	YES/NA/NA	NA	NO (but NLRP6)	NA	NO	NO (no cleavage)	NA	YES	(350)
Mouse BMDMs	poly(I:C)	Intracellular LTA	YES	NA (but in iBMDMs, YES)	YES	YES	YES/NA/NA	NA	NO (but NLRP6)	NA	NO	NO (no cleavage)	NA	YES	(350)
Young adult mice colonic (YAMC) epithelial cells	LPS	ATP	NA	NA	NO	NO	NO	YES	YES	NA	NA (but no PI intake)	YES (no cleavage)	NA	YES	(75)
Mouse BMDMs	LPS	PGN	NA	NA	NA	NA	NA	NA	NA	NA	NO	NA	NO	YES	(356)

NA, data not available.

### 5.2 Pyroptosis-predominant uncoupling

The most evident pyroptosis-predominant uncoupling is observed in NLRC4 inflammasomes in the absence of ASC. ASC deficiency in mouse BMDMs infected with *S. typhimurium*, *Pseudomonas aeruginosa*, or *L. pneumophila* inhibits the processing and secretion of IL-1β and caspase-1 (p20); LDH release is not impaired, whereas NLRC4 or caspase-1 deficiency abrogates all (358). The AIM2 inflammasome, which requires ASC for simultaneous cytokine secretion and pyroptosis, enables poly (dA:dT)-treated or *Francisella novicida*-infected iBMDMs to release LDH without IL-1β secretion and caspase-1 processing (p20) in an Asc KO setting when the NLRC4 CARD is fused onto the AIM2 sensor (358). Catalytical mutation or caspase-1 inhibition abolishes the ASC-independent death, whereas the auto-cleavage-deficient mutant caspase-1 D6N or C71 is still able to cleave GSDMD (25, 358). The NLRC4-caspase-1 complex, which is smaller than the ASC specks, is therefore termed the “death complex” or “death inflammasome”, downstream of which the pyroptosis-predominant uncoupling occurs without mature IL-1β secretion but potential pro-IL-1β release *via* lysis (222, 358). The newly emerged mechanism for caspase-1 activation and deactivation allows a putative explanation of the mysterious uncoupling events in these complexes compared to the coupling events in ASC specks (**Figures 4B, C**); both pyroptosis and IL-1β maturation are promoted in larger ASC specks when p33/p10 is the dominant species (in the NLRC4-ASC-caspase-1 complex). However, when p46 is the dominant species (in NLRC4-caspase-1 inflammasome in ASC^-/-^ macrophages), pyroptosis-predominant uncoupling can occur as p46 caspase-1 cleaves GSDMD but not pro-IL-1β (305). Therefore, caspase-1 dynamics, which can be mediated by ASC, play a key role in the death complex signaling. *L. pneumophila* induces cytotoxicity in human primary monocytes without IL-1β release, which is partly due to the decreased ASC expression in NLRC4 signaling (180). Additionally, while ASC^PYD^ mutations in the interface I and III impair their ability to trigger ASC specks and downstream events in NLRP3/AIM2/PYRIN signaling in iBMDMs, the Y59A or E80R mutations in the interface II maintain their capacity to trigger LDH release in the absence of speck formation and IL-1β secretion (222). These data pose an intriguing question as to how exactly ASC and its speck formation regulate IL-1β-free pyroptosis by modulating caspase-1 activity. NLRP3 activation in ASC^+/-^ settings also generates both p46 and p33/p10 active species; however, whether pyroptosis is balanced with cytokine processing remains unclarified (305). Further studies and more direct evidence may broaden the concept of the death complex into a specific caspase-1 activation model that facilitates pyroptosis under specific conditions.

However, whether another CARD-containing inflammasome sensor Nlrp1b could exhibit pyroptosis-predominant uncoupling preference in ASC-deficient settings remains controversial. Transduction of the functional Nlrp1b from 129S1 to C57BL/6 Asc^-/-^ macrophages results in increased LDH release with no IL-1β secretion when the cells are treated with anthrax lethal toxin (358). However, in response to the same toxin, the functional Nlrp1b (WT BALB/c and C57BL/6J [B6]^Nlrp1b^) induces simultaneous IL-1β secretion (detected in both supernatant and serum) and pyroptosis (indicated by LDH or HMGB1 release) in a caspase-1-dependent manner with no NLRP3, caspase-11, ASC expression, speck formation, or caspase-1 autoproteolysis (p20) required, both *in vitro* and vivo (310, 311). These data indicate that ASC specks may amplify the IL-1β release in Nlrp1b signaling, but are not necessarily required, depending on the differential genetic background, which differs from NLRC4 signaling, which acts as a death complex or induces coupling events in the absence or presence of ASC, respectively. Further studies are needed to explore how CARD-containing inflammasomes determine the downstream coupling or uncoupling preferences, especially in settings in which ASC specks do not form.

## 6 Discussion

**Figure 5:**
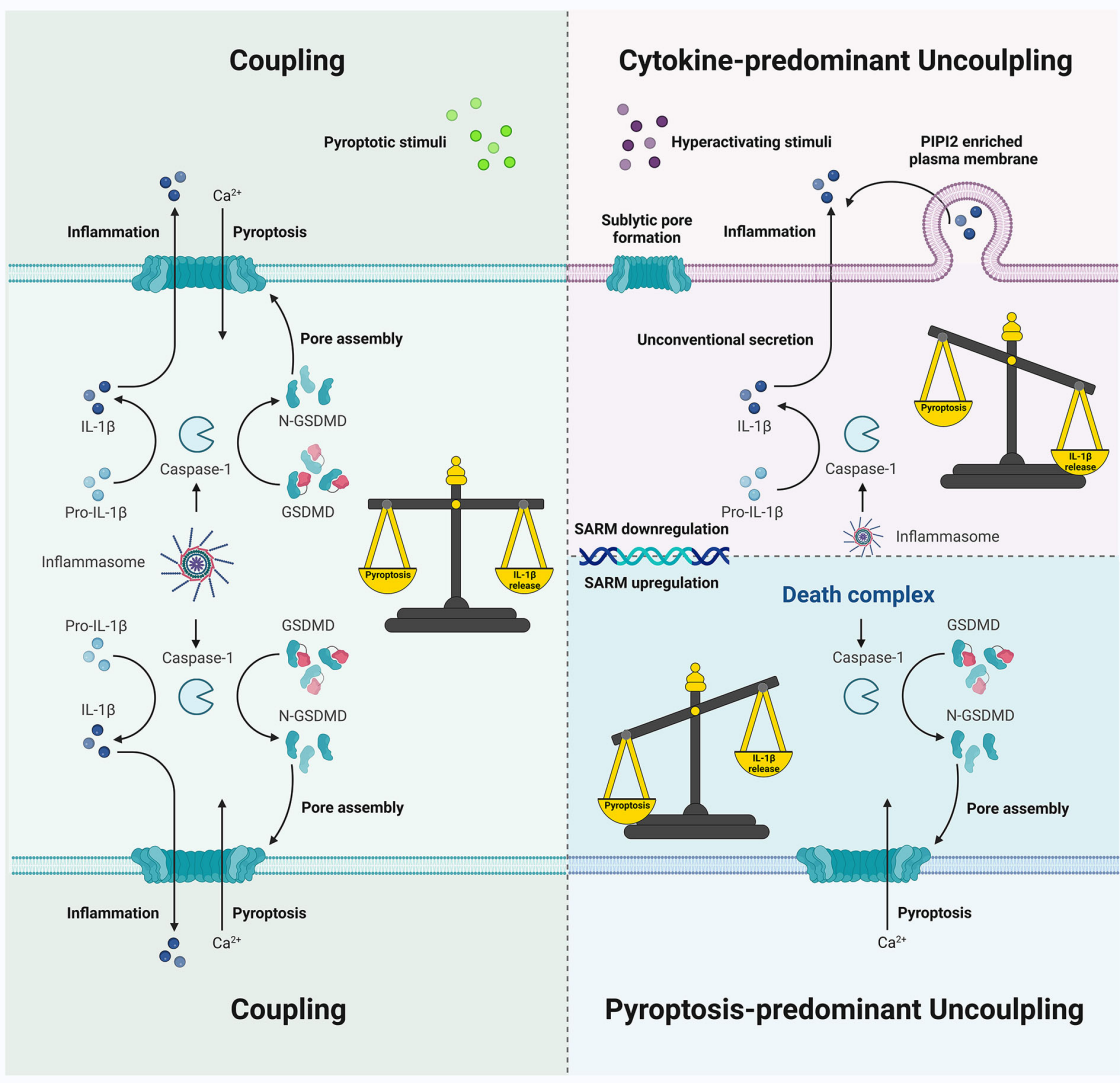
Schematic showing coupling and uncoupling events downstream of inflammasome signaling. Pyroptotic death and IL-1β secretion are commonly coupled as simultaneous consequences of inflammasome activation. Cytokine-predominant uncoupling is observed in hyperactivating stimuli challenges with mechanisms that depend on GSDMD pores or GSDMD molecules but not the pore formation on the plasma membrane; SARM deficiency may be involved in unconventional IL-1β secretion without pyroptosis in certain cell types. In contrast, death complex exhibits a pyroptosis-predominant uncoupling preference, which may be due to the differential behavior of active caspase-1 species during inflammasome activation.

The two major functional outcomes in inflammasome signaling are pyroptosis and cytokine secretion, which may be usually observed as coupled events (**Figure 5**). However, discrepancies among the diverse key steps in the signaling transduction include (1) caspase-1 processing, activation, and deactivation dynamics mediated by ASC specks (if not always) (2), GSDMD pore formation, pyroptotic cell death, and cell membrane rupture, and (3) IL-1β maturation and secretion in a GSDMD-dependent or -independent manner, or *via* cell lysis. These discrepancies may affect our understanding of the uncoupling events that occur downstream of inflammasome signaling. Besides possible misunderstandings, the underlying mechanisms of uncoupling remain elusive. Besides, the inflammasome sensor/ASC/caspase-1/GSDMD/IL-1β functions, the type and intensity of stimuli, the expression, and activation of SARM, other gatekeepers, participants, or executors of different levels, and the cell type and its microenvironment, all determine the occurrence of coupling or uncoupling and affect the direction in which specific uncoupling falls. Thus, subtle interrelations within inflammasome machinery and other non-inflammasome components exert significant effects on cell fate decisions. This raises an interesting question into why our immune system is so costly in terms of multilevel regulation of the coupling and uncoupling of pyroptosis and cytokine secretion, or why, how, and when a certain uncoupling appears to be preferred in some scenarios, since coupling is more commonly observed, at least *in vitro*, and is generally more rapid and robust when pyroptotic cells release DAMPs and IL-1β together as a massive inflammatory response.

There appear to be at least three reasons associated with uncoupling preferences. Firstly, hyperactivation and pyroptosis (even along with cytokine secretion) differ considerably in terms of host defense. Hyperactivation allows the living immune cells to add IL-1β and/or IL-18 into the cytokine repertoire, improving upon the repertoire of inflammatory mediators such as TNF-α and IL-6 in response to TLR agonists and resulting in prolonged pro-inflammatory effects on neighboring cells in situ, and even at distance *via* circulation. A well-characterized example is the salmonella infection in neutrophils. During acute salmonella-induced mouse peritonitis, resident macrophages secrete the first wave of IL-1β within 1 h post-infection, followed by rapid pyroptosis, and pyroptosis-resistant neutrophils are subsequently recruited as the main source of IL-1β 1–12 h post-infection (142). Neutrophil depletion increases the bacterial burden in mouse liver and spleen (142). These data suggest that the capacity of live neutrophils to fight pathogens is cytokine dependent. Additionally, hyperactive cells also contribute to shaping adaptive immunity by promoting T-cell differentiation and effector function (338). Hence, hyperactivation may initiate a modest and prolonged cytokine-mediated immunomodulatory effect, compared to the robust but relatively short (at least at a single-cell level) effects of pyroptosis coupled with cytokine secretion, and excessive hyperactivation may also be detrimental to the host defense (75, 350). In contrast, pyroptotic cell death, which can lead to massive inflammatory responses by exposing intracellular PAMPs and DAMPs, is so costly that dead cells cannot produce cytokine (359). The cost of inflammatory death at the single cell level may be compensated for by warning other cells to provide further protection against danger. However, the cost could be fatal at the host level; in some scenarios, cell pyroptosis (along with cytokine secretion) is a dangerous “signal” that is associated with *in vivo* lethality as compared to hyperactivation. LPS-primed mice all die from a second dose of LPS within 30 h, but sustain viability when challenged by oxPAPC or PGPC, indicating that stimuli (e.g., double LPS treatment) that trigger pyroptosis *in vitro* result in lethal sepsis *in vivo*, whereas the stimuli that induce hyperactivation in cells may promote inflammation without increased lethality in animal models (337). Sarm^-/-^ mice that are doubly challenged with LPS also exhibit an improved clinical score and survival compared to WT mice (356). These data help in further understanding two options: first, rapid death in a single cell or host to protect other cells and hosts and second, hyperactivation, wherein hyperactive cells secrete IL-1β and call for help from other cells to fight against DAMPs and PAMPs. Hence, the balance between benefit and cost needs to be carefully considered for the most appropriate decisions to be made in terms of cell/host fate.

Secondly, on a single cell level, hyperactivation enables delicate reactions to different stimuli at certain intensities in specific conditions. In terms of stimulus intensity, even the classic pyroptosis-inducer nigericin has been shown to promote hyperactivation in iBMDMs at relatively low doses (0.5 μM) as compared to higher doses (20 μM), affecting both IL-1β secretion and pyroptosis (53). In terms of stimuli type and microenvironmental context, oxPAPC alone (without priming) induces CD14 deficiency in cell membranes and inhibits its binding to LPS and the TLR4 signaling, whereas LPS-primed and oxPAPC-treated DCs become hyperactive (337). The former may imply sterile inflammation in which DAMPs alone are detected, and a less sensitive response to PAMPs is therefore subsequently required to avoid excessive autoinflammation; however, if the immune system detects PAMPs followed by DAMPs, a dangerous infection is implied, which results in inflammatory hyperactivation to cope with virulent pathogens and tissue damage. Notably, these differential reactions are also organized in a cell type- and species-dependent manner. oxPAPC induces robust IL-1β secretion from GMCSF-DCs, and to a lesser extent from FL3TL-DCs (338), and can also exert a cytotoxic effect on cDC1 but not cDC2, whereas PGPC does not induce the release of LDH in either population (338). These data suggest the differential effects of hyperactivating stimuli on DCs in different subgroups. In addition, as the proteins CD14 and TLR4 are important in LPS internalization, LPS alone activates the alternative inflammasome signaling in human monocytes that express abundant CD14, whereas IL-1β secretion is not detected in LPS-treated murine PBMCs or human macrophages and DCs with lower CD14 expression unless a second signal occurs to induce the simultaneous release of LDH and IL-1β (343, 346). It is conceivable for monocytes to be more sensitive than macrophages or DCs under hyperactivation as a result of the recruitment and differentiation of monocytes in the blood into macrophages and DCs in LPS concentration-rich bacterial infection sites; the rapid activation of live monocytes with IL-1β secretion could act as an acute response in the frontline against pathogens. Another cell type-dependent example is pyroptosis-resistant neutrophils. Human neutrophils, which have a relatively short life span (< 1 day), require timely replacement and efficient anti-bacterial actions, rendering anti-pyroptosis a considerate strategy (360). Neutrophils have lower GSDMD mRNA and p30 fragment expression, with less caspase-1 and ASC levels and smaller specks than macrophages and DCs, facilitating longer caspase-1 activity IL-1β secretion (12, 39, 52, 305, 352, 355). While macrophages undergo caspase-1-dependent pyroptosis to prevent intracellular *S. Typhimurium* replication and raise further anti-bacterial responses from neighboring cells (e.g., more effective killing by recruited neutrophils), caspase-1 deficiency has no effects on the intracellular bacterial burden of neutrophils, further suggesting that these cells may adopt a strategy other than pyroptotic lysis in canonical inflammasome signaling (142, 359). In contrast, one may expect that neutrophils committing inflammatory suicide utilize a rapid way to kill intracellular pathogens over the short-term instead of pyroptosis resistance. Indeed, pyroptosis-resistant neutrophils do not promote macrophage efferocytosis, which enables macrophages to engulf dying neutrophils, rendering pyroptosis resistance an unsatisfactory means of building an immune response (355). However, pyroptosis exemption could extend the lifespan of neutrophils, allowing for degranulation, reactive oxygen species (ROS) production, chasing and killing bacteria such as those released by pyroptotic macrophages, and recruiting more neutrophils to the infection site due to the consistent direct and indirect GSDMD-dependent IL-1β secretion (352, 361). While pyroptosis-resistant neutrophils secrete comparable levels of IL-1β when primed with LPS and treated with ATP as compared to macrophages, IL-1β release is further increased in neutrophils but significantly decreased in macrophages when cells are pre-treated with injured cell-derived supernatants or extracellular ATP, further suggesting the significance of neutrophils as an important IL-1β source in the danger signal-enriched milieu (355). Therefore, by regulating coupling and uncoupling within the cell population, a highly effective cooperation and amplification system can be organized with each component involved in both anti-infection and anti-danger functions. Notably, the exact role of GSDMD-NT, in classic NETOsis (which is supposed to trap extracellular pathogens) is contradictory; however, under cytosolic Gram-negative bacteria infection, caspase-11/GSDMD-mediated noncanonical NETosis would protect neutrophils from bacterial invasion and decrease the intracellular bacterial burden under GSDMD cleavage and IL-1β secretion (39, 43, 44). Furthermore, whether and how neutrophiles balance or “unbalance” pyroptosis and IL-1β release may be influenced by host species, readout timing, priming requirement, and notably the level of neutrophiles granules that may act as “sinks” to capture GSDMD-NT and avoid pyroptosis (349). Hence, these multi-layered anti-bacterial strategies allow neutrophils to behave versatilely in a context-dependent manner. Hyperactivation may also occur in nonimmune cells. Caspase-1 dimerization induces IL-1β release in viable immortalized mouse embryonic fibroblasts without rupturing the cell membrane (312). A possible explanation is that some nonimmune cells do not endogenously express inflammasome components for massive GSDMD pore formation (312). Likewise, pyroptosis-predominant uncoupling is also tailored to specific conditions where an inflammasome without an ASC focus is achieved under experimental conditions (e.g., NLRC4-caspase-1 complex in ASC^-/-^ macrophages), mutations (e.g., ASC^Y59A^ and ASC^E80R^ in NLRP3/AIM2/PYRIN signaling), and infections, and is also dependent on cell type (neutrophils are an exception with smaller ASC specks for p46 and p33/p10 species accumulation but with poor pyroptotic modalities and dynamics, as mentioned previously). Since different immune/nonimmune cells and their differential subgroups are responsible for multiple distinct and overlapping (if any) roles in building innate and shaping adaptive immunity, the alternative strategies of coupling or uncoupling are supposed to function in a specific manner. The fate decision is made not only on the single cell level but also in the cell population within the microenvironment, where different and associated cells cooperate to cope with intricate challenges, foreign or endogenous. Therefore, further *in vivo* studies are required to clarify how the cell players work together as a whole if different strategies are preferentially chosen by different subgroups.

Finally, uncoupling may be adopted by bacteria or host to fight against each other in different situations. From the bacterial perspective, as some Gasdermin family members are also found in fungi and bacteria, their pore structures may function as transport systems that allow microbes to release proteins into the periplasm or extracellular space (55). This hypothesized mechanism is designed so that unreversible cell death is not triggered, even in host cells. In this way, bacteria (e.g., *S. Typhimurium*) may evade neutrophil-induced death by avoiding pyroptotic-mediated disruption of the intracellular niche, at least in the short term. Therefore, from the host’s perspective, the prolonged IL-1β secretion is induced by caspase-1 activation following ASC speck formation to fight bacteria. However, bacterial infection may also regulate the host’s defense by utilizing pyroptosis. *L. pneumophila* infection inhibits NLRC4 and ASC expression in human monocytes to avoid robust IL-1β secretion, and the innate immune system has the other means of fighting back; the relatively inactive NLRC4 inflammasome with less ASC participants is still able to induce cell death while caspase-1 remains unprocessed, however, the intracellular bacteria count may still increase in the cytotoxic background (180). In this situation, caspase-7 (in a GSDMD-independent manner) and the GSDMD are responsible for pore formation-induced cell death and the restriction of bacterial replication in response to *L. pneumophila* infection, both *in vivo* and *in vitro*, indicating the significance of cell death in the anti-bacterial response (204, 362, 363). However, as mentioned previously, excessive pyroptosis is dangerous to hosts. In some scenarios, pyroptosis may lead to bacteria (e.g., Mtb) spreading to adjacent healthy cells as new hosts, facilitating replication (364). Pyroptosis (and the backup pathways of apoptosis, if any) may even promote mouse death as a result of NLRC4 overactivation in response to non-propagative *S. Typhimurium*, indicating that inflammasome-induced damage is lethal, whereas bacterial burden is not, although the propagation of WT strains is hampered by inflammasome signaling (e.g., IL-1β secretion in hyperactive neutrophils/monocytes and pyroptotic macrophages) (142, 344, 365). The balance between pathogen and host renders our understanding of inflammasome signaling and downstream events complex, where the very same strategy (e.g., pro-pyroptosis or anti-pyroptosis) may dynamically benefit one side in some situations, and the other in others.

Despite these possible reasons, more detailed answers are required to clarify the exact mechanisms, timing, and regulation of coupling and uncoupling, especially the poorly understood roles of the uncommonly localized inflammasome components (e.g., extracellular ASC specks), non-inflammasome participants, the connection between and pyroptosis and other forms of regulated cell death, and other inflammatory pathways and inflammasome activation mechanisms. Uncoupling downstream of inflammasome signaling has an outstanding role in homeostasis and host defense and is thus attracting increasing interest, rendering this field worthy of further exploration. However, more attention should be paid to the design of lab investigations. For example, because GSDMD-mediated pyprotosis and cytokine secretion are observed using numerous readouts, the accurate interpretation of the meaning of such readouts is important. Although the mechanisms surrounding glycine and punicalagin are unclear, the effects of the two cytoprotectants may differ. Punicalagin inhibits the release of IL-1β and LDH at similar half-maximal inhibitory concentrations (IC50) of 3.91 and 3.67 μM in ATP-treated macrophages, whereas glycine prevents the release of LDH (not in THP-1 cells) but not IL-1β secretion or PI intake, suggesting different roles of blocking plasma membrane permeabilization and rupture, respectively (20, 52, 68). PI uptake may always be detected alongside IL-1β secretion, although in some scenarios the latter is observed without the former, perhaps because of the subthreshold GSDMD pore formation in hyperactive cells or the partially indirect role of GSDMD-NT in neutrophil IL-1β release (52, 344, 352, 353). In contrast, LDH release is more likely considered a result of cell membrane rupture, although controversial data shows that glycine cannot inhibit the release of LDH from pyroptotic THP-1 cells (20). The evaluation of LDH release may be used in combination with other means that assess cell viability to further conclude hyperactivation in IL-1β secreting cells. Moreover, since LDH release could indicate other forms of cell membrane rupture, attention should be paid when using it as a sole marker for pyroptosis (39). Additionally, the possibility of detecting extracellular pro-IL-1β using ELISA should be considered to avoid the incorrect detection of IL-1β (68). More importantly, as the relative responses are unique in each species, the interpretation and extrapolation of data from animal models should be carefully considered when searching for potential targets or developing novel therapies for human diseases. Collectively, studies investigating the downstream events of inflammasome signaling should be carefully designed to consider species, cell type, and readouts to avoid misunderstanding.

Furthermore, when considering pathological conditions, uncoupling can be protective or detrimental to the host, and excessive or inadequate activation may backfire in the initial attempt to maintain homeostasis. Hyperactive-like neutrophils are involved in the pathogenesis and development of autoinflammatory disorders (e.g., cryopyrin-associated periodic syndromes [CAPS]) in gain-of function models (366, 367), and hyperactivating stimuli may also lead to inflammation but not death in mouse sepsis (337). Pyroptotic death and its backup apoptosis may help to limit bacterial reproduction in cells and tissues but may be lethal to mice under overactive inflammasome signaling (365). “Incompetent” uncoupling could be too weak to control bacterial replication or too strong to avoid massive and widespread damage that may threaten host survival (368). Hence, when developing potential therapies for uncoupling-associated pathological conditions, the use of pro-uncoupling, coupling, or inhibition of inflammasome activation should be carefully balanced to consider the benefits and costs. As multicellular hosts possess a sophisticated network of differential and cooperative cellular and molecular players, therapies need to be balanced so that any deleterious effects can be limited on all components. Further studies that shed light on the roles of uncoupling events downstream of inflammasome signaling may thus create promising opportunities for novel drug development.

## Author contributions

YL wrote the manuscript and created the figures. QJ reviewed the manuscript and provided guidance. Both authors contributed to the article and approved the submitted version.
